# Integrative analysis of the gut microbiota and faecal and serum short-chain fatty acids and tryptophan metabolites in patients with cirrhosis and hepatic encephalopathy

**DOI:** 10.1186/s12967-023-04262-9

**Published:** 2023-06-17

**Authors:** Qiang Wang, Chengxin Chen, Shi Zuo, Kun Cao, Haiyang Li

**Affiliations:** 1 Department of Hepatobiliary Surgery, The Affiliated Hospital of Guizhou Medical University, Guiyang, Guizhou People’s Republic of China; 2 School of Clinical Medicine, Guizhou Medical University, Guiyang, Guizhou People’s Republic of China

**Keywords:** Hepatic encephalopathy, Liver cirrhosis, Gut microbiota, Short-chain fatty acids, Tryptophan

## Abstract

**Objective:**

The purpose of this study was to describe the changes in the gut microbiome of patients with cirrhosis and hepatic encephalopathy (HE), as well as quantify the variations in short-chain fatty acid (SCFA) and tryptophan metabolite levels in serum and faeces.

**Methods:**

Fresh faeces and serum were collected from 20 healthy volunteers (NC group), 30 cirrhosis patients (Cir group), and 30 HE patients (HE group). Then, 16S rRNA sequencing and metabolite measurements were performed using the faeces. Gas chromatography‒mass spectrometry and ultrahigh-performance liquid chromatography-tandem mass spectrometry were used to measure SCFA and tryptophan levels, respectively. The results were analysed by SIMCA16.0.2 software. Differences in species were identified using MetaStat and t tests. The correlations among the levels of gut microbes and metabolites and clinical parameters were determined using Spearman correlation analysis.

**Results:**

Patients with cirrhosis and HE had lower microbial species richness and diversity in faeces than healthy volunteers; these patients also had altered β-diversity. Serum valeric acid levels were significantly higher in the HE group than in the Cir group. Serum SCFA levels did not differ between the Cir and NC groups. Serum melatonin and 5-HTOL levels were significantly higher in the HE group than in the Cir group. The Cir and NC groups had significant differences in the levels of eight serum tryptophan metabolites. Furthermore, the levels of faecal SCFAs did not differ between the HE and Cir groups. Faecal IAA-Ala levels were significantly lower in the HE group than in the Cir group. There were significant differences in the levels of 6 faecal SCFAs and 7 faecal tryptophan metabolites between the Cir and NC groups. Certain gut microbes were associated with serum and faecal metabolites, and some metabolites were associated with certain clinical parameters.

**Conclusion:**

Reduced microbial species richness and diversity were observed in patients with HE and cirrhosis. In both serum and faeces, the levels of different SCFAs and tryptophan metabolites showed varying patterns of change. In HE patients, the levels of some serum tryptophan metabolites, and not SCFAs, were correlated with liver function and systemic inflammation. Systemic inflammation in patients with cirrhosis was correlated with faecal acetic acid levels. In summary, this study identified metabolites important for HE and cirrhosis.

**Supplementary Information:**

The online version contains supplementary material available at 10.1186/s12967-023-04262-9.

## Introduction

Liver cirrhosis is a serious type of chronic liver disease that often results in high rates of morbidity and mortality [1]. Liver-related complications, such as hepatic encephalopathy (HE), which can affect multiple organs outside the liver, are the primary cause of death among individuals with this condition [2]. HE is a neuropsychiatric disorder that is associated with acute or chronic liver disease and is characterized by varying degrees of cognitive and fine motor impairments, such as inattentiveness, blunted affect, impaired memory, reversed sleep-wake cycle, tremors, myoclonus, asterixis, and deep tendon hyperreflexia [3]. Epidemiological studies have revealed that the prevalence of HE is increasing globally [4]. As a result, it is crucial to prevent or treat HE as early as possible to enhance prognosis. However, the pathogenesis of HE is a complex and poorly understood process that includes hyperammonaemia, inflammation, changes to the gut microbiota, oxidative stress, mitochondrial dysfunction, and neurotransmitter abnormalities [3]. Unfortunately, no single factor has been shown to fully explain the pathogenesis of HE, which leads to poor treatment outcomes and prognosis.

Ammonia and inflammation are key factors involved in the pathophysiologic development of HE [5]. Recent studies have shown that patients with cirrhosis have an imbalanced intestinal flora [6, 7]. These changes can lead to the excessive production of ammonia, resulting in hyperammonaemia [8]. Additionally, alterations in the gut microbiota have been shown to promote the production of endotoxins, leading to both systemic and neurological inflammation [9]. These findings suggest a strong correlation between alterations in the gut microbiota and the onset of HE in individuals with cirrhosis. Several previous studies have also reported a notable alteration in the microbiota structure among patients with HE [10, 11]. However, importantly, the function and effects of bacteria can vary greatly based on the phyla or family.

In patients with cirrhosis and HE, the gut microbiota undergoes alterations, and there is also a change in the shared metabolic processes that occur between the host and microbiota [10]. Research has shown that patients with HE exhibit a decline in short-chain fatty acid (SCFA) levels in their intestinal tract due to a reduction in the levels of SCFA-producing species (such as *Anaerostipes caccae*) [12]. Low levels of SCFAs can contribute to the development of HE by enhancing the transfer of neurotoxins, weakening intestinal barrier function, and increasing intestinal barrier permeability [13, 14]. Furthermore, a correlation has been observed between reduced serum SCFA levels and cirrhosis [6]; however, different SCFAs serve various functions [15, 16]; for example, butyrate primarily targets colonocytes to generate energy, while propionate is mainly transported to the liver, where it promotes gluconeogenesis [17]. Only butyrate exhibits significant epigenetic properties that are involved in various diseases [18]. Understanding the clinical importance of SCFAs by assessing alterations in their levels may provide a new perspective for understanding the pathogenesis of HE and may provide more options for its treatment. Nevertheless, few studies have explored the relationship between both faecal and serum SCFAs and cirrhosis or HE to date.

Additionally, certain metabolites of tryptophan, namely, indoleacetic acid (IAA) and indolepropionic acid (IPA), have been shown to impact intestinal permeability and immune function [19, 20]. A recent study suggested that alterations in tryptophans may contribute to the development of inflammatory bowel disease, obesity, and neuropsychiatric disorders [21]. The liver plays a crucial role in the metabolism of tryptophan, and any disruptions in this process can result in fluctuations in the levels of its metabolites. These fluctuations can potentially impact the functioning of the central nervous system. As HE has neurological and psychiatric symptoms, tryptophan metabolites may also be closely related to HE. Nevertheless, there is currently no research exploring the connection between tryptophan metabolites and cirrhosis or HE.

In the context of the information presented above, our study aimed to provide a comprehensive description of the alterations in the gut microbiome of individuals with cirrhosis or HE and to quantitatively evaluate the alterations in the levels of SCFAs and tryptophans in both serum and faeces. Additionally, we explored the correlation between the gut microbiota and these two types of metabolites, the relationship between the levels of these two types of metabolites in the serum and faeces of cirrhosis and HE patients, and their correlation with clinical parameters.

## Materials and methods

### Research subjects, selection criteria, and exclusion criteria

Healthy volunteers (NC group), overt HE (OHE) patients (HE group), and patients with cirrhosis but without HE (Cir group) were recruited at the Affiliated Hospital of Guizhou Medical University (Guizhou Province, China) from January to December 2022. Written consent was provided by all participants before the collection of specimens. Faeces and blood samples were collected from all participants to characterize the gut microbiota, faecal metabolites (SCFAs and tryptophans), and serum metabolites (SCFAs and tryptophans). Healthy volunteers were selected based on the following assessments: medical history, physical examination, chest X-ray, routine blood tests, blood glucose, liver function, kidney function, and other physical and chemical examinations, which revealed no diseases of the brain, heart, lung, liver, kidney, or other major organ systems. A liver biopsy, the discovery of varices or portal hypertension, or the decompensation of chronic liver disease were all used to diagnose liver cirrhosis [22]. HE was diagnosed according to the 2014 AASLD/EASL HE guidelines [23]. OHE was determined to be Grade II or higher HE based on features such as disorientation, somnolence, and coma-like symptoms [3]. Patients with diabetes, mental illness, hepatocellular carcinoma, primary sclerosing cholangitis, autoimmune hepatitis, gastrointestinal diseases, and a history of gastrointestinal surgery were excluded. Additionally, patients who underwent hormone replacement therapy or used antipsychotic medication, antibiotics, or probiotics within the 6 weeks prior to the study, as well as patients with active alcohol use disorder (AUDIT-10 > 8), were excluded [22]. This study was conducted in accordance with the guidelines of Helsinki’s Declaration and received approval from the Medical Ethics Committee of Guizhou Medical University (Ethics No. 2020007).

### Sample collection and processing

Fresh faeces (200–300 mg) from patients and volunteers were collected into two separate disposable sterile 5 mL tubes. One tube was used to sequence the gut microbiota, and the other was used to detect metabolites. Each sterile tube was labelled, placed in an ice box, and then transferred to the laboratory. Subsequently, specimens used for the detection of metabolites were frozen in liquid nitrogen for one minute before being kept at − 80 °C. The next morning, peripheral venous fasting blood from each participant was drawn and collected in tubes that were then labelled. Blood was then centrifuged to obtain the serum, and 0.5 mL of serum was placed in 1.5 mL centrifuge tubes. Before usage, the sera sample tubes were labelled and kept at − 80 °C. Clinical information was obtained from patients, and basic information was obtained from volunteers.

### Targeted metabolomics study

#### Short-chain fatty acids

##### Extraction of serum metabolites

A 100 µL serum sample along with two internal standards (0.2 mL of 2-methylpentanoic acid and 0.05 mL of 50% H_2_SO_4_) were put into an Eppendorf (EP) tube. The mixture was shaken for 10 min, spun for 30 s, and then sonicated in an ice bath for 10 min. Following a 15-min centrifugation at 10,000 rpm, the mixture was kept at − 20 °C for 30 min. The supernatant was collected for gas chromatography‒mass spectrometry (GC‒MS).

##### Extraction of faecal metabolites

A 100 mg faecal sample diluted in 1 ml of distilled water was placed in an EP tube, vortexed, shaken, homogenized, and sonicated. Subsequently, the material was centrifuged at 5000 rpm for 20 min. Then, 0.8 mL of the supernatant, 0.1 mL of H_2_SO_4_ (50%), and 0.8 mL of 2-methylpentanoic acid were added to the tube, followed by vortexing and sonication. The sample was kept at − 20 °C for 30 min after being centrifuged for 15 min at 10,000 rpm. The supernatant was finally collected for GC‒MS analysis.

##### GC‒MS analysis

The GC-mass spectrometer used for the GC‒MS analysis was a SHIMADZU GC2030-QP2020 NX. GC‒MS analysis followed the protocols of previous work [24]. All equipment information and the detailed methodology for GC‒MS are available in the Additional file 3: Methods.

### Tryptophan

#### Extraction of serum metabolites

A 100 µL serum sample and 400 µL of extract solution were placed in an EP tube, vortexed, shaken, and sonicated before being kept at − 40 °C for an hour. A new EP tube was used to collect 400 µL of the supernatant after centrifugation (12,000 rpm; 15 min). This supernatant was then dried in nitrogen and redissolved in 100 µL of an aqueous solution containing 0.1% formic acid. To recover the supernatant for ultrahigh-performance liquid chromatography-tandem mass spectrometry (UHPLC‒MS/MS) analysis, the supernatant was centrifuged at 12,000 rpm for 15 min.

#### Extraction of faecal metabolites

A 50 mg faecal sample was mixed with 500 µL extract solution in an EP tube. The mixture was vortexed, shaken, homogenized, sonicated, and allowed to sit at -40 °C for 1 h. Then, 320 µL of supernatant was transferred to a fresh EP tube, dried in nitrogen, and redissolved in an 80 µL volume of aqueous solution. The supernatant was centrifuged and collected for UHPLC‒MS/MS analysis.

#### UHPLC-multiple reaction monitoring (MRM)-MS analysis

The target compounds were separated on a Waters ACQUITY UPLC HSS T3 liquid chromatography column using an EXIONLC System (SCIEX) (Sciex, Framingham, MA, USA). UHPLC-MRM-MS analysis was performed according to previous studies [25, 26]. All equipment information and software and the detailed methodology for UHPLC-MRM-MS are available in the Additional file 3: Methods

#### DNA extraction, 16S rRNA amplification, and sequencing

Using the CTAB/SDS technique, total genomic DNA was isolated from the samples [27]. Using 515 F-806R primers, the 16 S rRNA genes in various locations were amplified [28]. The amplification of 16S rRNA and the quality assessment of polymerase chain reaction products were performed following the protocol of a previous study [29]. Illumina sequencing libraries were created using the NEBNext^®^ UltraTM IIDNA Library Prep Kit (Cat No. E7645). The quality of the libraries was assessed using a Qubit@ 2.0 fluorometer from Life Technologies GmbH in Darmstadt, Germany, and an Agilent Bioanalyzer 2100 system from Agilent Technologies in Waldbronn, Germany. The libraries were sequenced on the Illumina NovaSeq platform in accordance with the manufacturer’s instructions (Illumina Inc., San Diego, USA).

#### Follow-up study

Telephone conversations with patients or members of their immediate families were conducted for follow-up. The follow-up period was 90 days after discharge. The endpoints of this study comprised mortality associated with cirrhosis or HE, recurrence, and readmission related to cirrhosis or HE.

### Statistical analysis

#### Clinical parameters

SPSS 26.0 software was used to conduct a statistical analysis of clinical parameters. A normality test was first performed on continuous variables. Quantitative normal variables are presented as the mean ± SD. The Wilcoxon rank-sum test was used to compare variables with uneven variance, whereas the t test was used to compare data with normal variance homogeneity. When comparing groups, the Wilcoxon rank-sum test was utilized, and the median was used to present nonnormal variables. A chi-square test was used to compare groups, and the grade values are reported as a frequency in percentages. A statistically significant difference was considered when the P value was lower than 0.05.

#### Sequencing data analysis

Initial amplicon sequence variations (ASVs) were denoised using QIIME2 software (version QIIME2-202006), and ASVs with an abundance of less than 5 were filtered out [30]. Taxonomic assignment was also performed using QIIME2. Using a sequence-number standard based on the sample with the fewest sequences, the absolute abundance of the ASVs was normalized. The generated normalized data were used to calculate the diversity indices. Chao1, Shannon, and Simpson were used to evaluate the diversity, while Pielou’s evenness index was employed to evaluate the diversity, richness, and consistency of the microbial community. Based on weighted and unweighted UniFrac distances, the β diversity was estimated.

Using principal component analysis (PCA), a cluster analysis was performed. To visualize the differences in the complicated multidimensional data between samples, principal coordinate analysis (PCoA) was performed. To overcome the constraints of linear models (PCA and PCoA), nonmetric multidimensional scaling (NMDS) based on the Bray–Curtis distance was carried out. To examine variations in community structure between groups, the adonis and anosim functions in QIIME2 were employed. Identification of species with significant differences in each classification level was performed using MetaStat and t tests in R software v3.5.3. Identification of biomarkers was performed using LEfSe analysis (LDA score = 4) with LEfSe software v1.0 [31]. Additionally, functional annotation analysis using PICRUSt2 software version 2.1.2-b [32] was carried out to determine the variations in community function between various groups.

#### Targeted metabolomics data analysis

Multivariate data containing the sample name, the compound name, and concentration details were analysed using SIMCA v16.0.2 software (Umetrics, Malmo, Sweden). To reduce noise and significant variations in the variables, the data were scaled and logarithmically transformed. After these adjustments, PCA was used to show the distribution and categorization of the samples. Outliers in the dataset were determined by using the 95% confidence interval in the PCA score plot. By using an orthogonal partial least squares discriminant analysis (OPLS-DA), the model’s dependability was assessed, and its evaluation indices (R2 and Q2) were determined by running seven cross-validations. The list of 31 tryptophan metabolites and their abbreviations are summarized in Additional file 1: Table S5.

#### Spearman correlation analysis

The correlation between the levels of gut microbes, SCFAs, and tryptophans and between these two types of metabolites and clinical parameters was determined using SPSS 26.0. The correlations between these features was visualized using the *pheatmap* package in R software.

## Results

### Characterization of participants

Initially, 106 specimens were collected from 86 patients and 20 healthy volunteers. According to our exclusion criteria, 21 patients were excluded, and five patients were not assessed. Finally, the entire sample size was 80 subjects, including 30 HE patients (HE group), 30 cirrhosis patients (Cir group), and 20 healthy volunteers (NC group). The HE, Cir, and NC groups differed significantly in age (*p* < 0.001). The ages in both the HE group (56.13 ± 13.20) and the Cir group (52.23 ± 9.00) were higher than that in the NC group (44.45 ± 8.46). The difference between the HE and Cir groups, however, was not statistically significant (p = 0.161). Notably, the three groups did not differ significantly in sex, body mass index (BMI), or aetiology (*p* = 0.888, 0.900, and 0.566, respectively). Patient Child‒Pugh scores were lower in the Cir group than in the HE group (p = 0.005). A higher model for end-stage liver disease score (Meld-score) was observed in the HE group (19.67 ± 5.96) than in the Cir group (13.90 ± 6.51). The HE group (70%) had a greater prevalence of moderate to severe ascites than the Cir group (26.67%). The rate of previous variceal haemorrhage was higher in the HE group (43.33%) than in the Cir group (16.67%). The HE group (43.33%) had a higher prevalence of transjugular intrahepatic portosystemic shunts (TIPS) than the Cir group (6.67%). A total of 36.67% of the participants in the HE group had spontaneous bacterial peritonitis, compared to 10.00% in the Cir group. The HE group had higher total bilirubin (TBIL), direct bilirubin (DBIL), indirect bilirubin (IBIL), prothrombin time (PT), international normalized ratio (INR), and ammonia levels than the Cir group, whereas alanine transaminase (ALT) and aspartate transaminase (AST) levels were lower in the HE group. There were no statistically significant differences between the proportions of 90-day mortality and 90-day recurrence in the HE group and the Cir group (13.33% and 16.67%, vs. 0% and 0%, respectively) (Table 1).

**Table 1:** Clinical parameters and laboratory test indexes of the study cohort

Variables	HE group (N = 30)	Cir group (N = 30)	NC group (N = 20)	*F/χ* ^*2*^ */t*	*P*
Age (year)	56.13 ± 13.20	52.23 ± 9.00	44.45 ± 8.46	7.245(*F)*	0.001 0.161^ab^ < 0.001^ac^ 0.015^bc^
Gender (Male/female)	19/11	20/10	12/8	0.234(*χ*^*2*^)	0.889
BMI	22.53 ± 2.51	22.21 ± 3.24	22.37 ± 2.07	0.106(*F*)	0.900
Etiology					
HBV HCV Alcoholic HBV + alcoholic HCV + alcoholic Others	9 1 12 1 1 6	14 1 8 3 1 3		3.887(*χ*^*2*^)	0.566
Child-Pugh Class					
A B C	0 9 21	9 7 14		10.650(*χ*^*2*^)	0.005
Meld-score	19.67 ± 5.95	13.90 ± 6.51		3.582(*t*)	< 0.001
Ascites					
None Mild Moderate Severe	2 7 8 13	15 7 2 6		16.120(*χ*^*2*^)	< 0.001
Medication history					
Non-selective β-blockers Lactulose Anti-HBV	4(13.33%) 5(13.33%) 9(30.00%)	0(0%) 0(0%) 15((60.00%)		Fisher’s Exact Test Fisher’s Exact Test 2.5(*χ*^*2*^)	0.112 0.052 0.114
Previous variceal bleeding	13(43.33%)	5(16.67%)		5.079(*χ*^*2*^)	0.024
Previous TIPs	13(43.33%)	2(6.67%)		10.756(*χ*^*2*^)	0.001
Previous HE	6(20.00%)	0(0%)		Fisher’s Exact Test	0.024
SBP	11(36.67%3)	3(10.00%)		4.565(*χ*^*2*^)	0.033
Creatinine (μmol/L)	74.2(53.95-156.15)	72.35(55.98–84.85)		Mann-Whitney U Test	0.487
TBIL (μmol/L)	51.91(27.96–94.53)	28.19 (14.42–56.31)		Mann-Whitney U Test	0.012
DBIL (μmol/L)	24.14 (12.84–59.47)	12.98 (6.36–36.20)		Mann-Whitney U Test	0.037
IBIL (μmol/L)	22.73 (11.12–45.37)	14.06 (7.54–22.03)		Mann-Whitney U Test	0.023
ALT (IU/L)	24.75(15.43–43.13)	44.40 (26.30-60.53)		Mann-Whitney U Test	0.006
AST(IU/L)	41.90 (26.88–68.55)	68.30 (47.35–92.48)		Mann-Whitney U Test	0.030
Albumin(g/L)	29.47 ± 4.64	31.76 ± 7.44		-1.428(*t*)	0.159
PT(s)	18.85(16.20–22.50)	16.55(13.97–18.50)		Mann-Whitney U Test	0.011
INR	1.63 (1.38–2.06)	1.38 (1.10–1.60)		U Test	0.004
Ammonia(μmol/L)	92.31 ± 47.51	41.51 ± 18.30		5.465(*t*)	< 0.001
WBC(10^9^/L)	6.10 ± 4.67	5.20 ± 2.16		0.965(*t*)	0.339
Neutrophil(%)	68.70 ± 13.24	65.06 ± 11.22		1.148(*t*)	0.256
CRP	6.54(2.26–18.42)	7.38(1.27-134.98)		Mann-Whitney U Test	0.813
Length of stay (days)	8.50(6.00-15.75)	8.50(7.00-15.50)		Mann-Whitney U Test	0.772
90-day mortality	4(13.33%)	0(0%)		Fisher’s Exact Test	0.112
90-day recurrence	5(16.67%)	0(0%)		Fisher’s Exact Test	0.052

*BMI* body mass index, *HBV* hepatitis B virus, *HCV* hepatitis C virus, *Anti-HBV* Anti-hepatitis B virus therapy, *TIPs* transjugular intrahepatic portal-systemic, *HE* hepatic encephalopathy, *SBP* spontaneous bacterial peritonitis, *TBIL* total bilirubin, *DBIL* direct bilirubin, *IBIL* indirect bilirubin, *ALT* alanine transaminase, *AST* aspartate transaminase, *PT* prothrombin time, *INR* international normalized ratio, *WBC* white blood cell count, *CRP* C-reactive protein. ab, difference between HE group and Cir group; ac, difference between HE group and NC group; bc, difference between Cir group and NC group

### Altered gut microbiota diversity in cirrhosis and HE patients

**Fig. 1:**
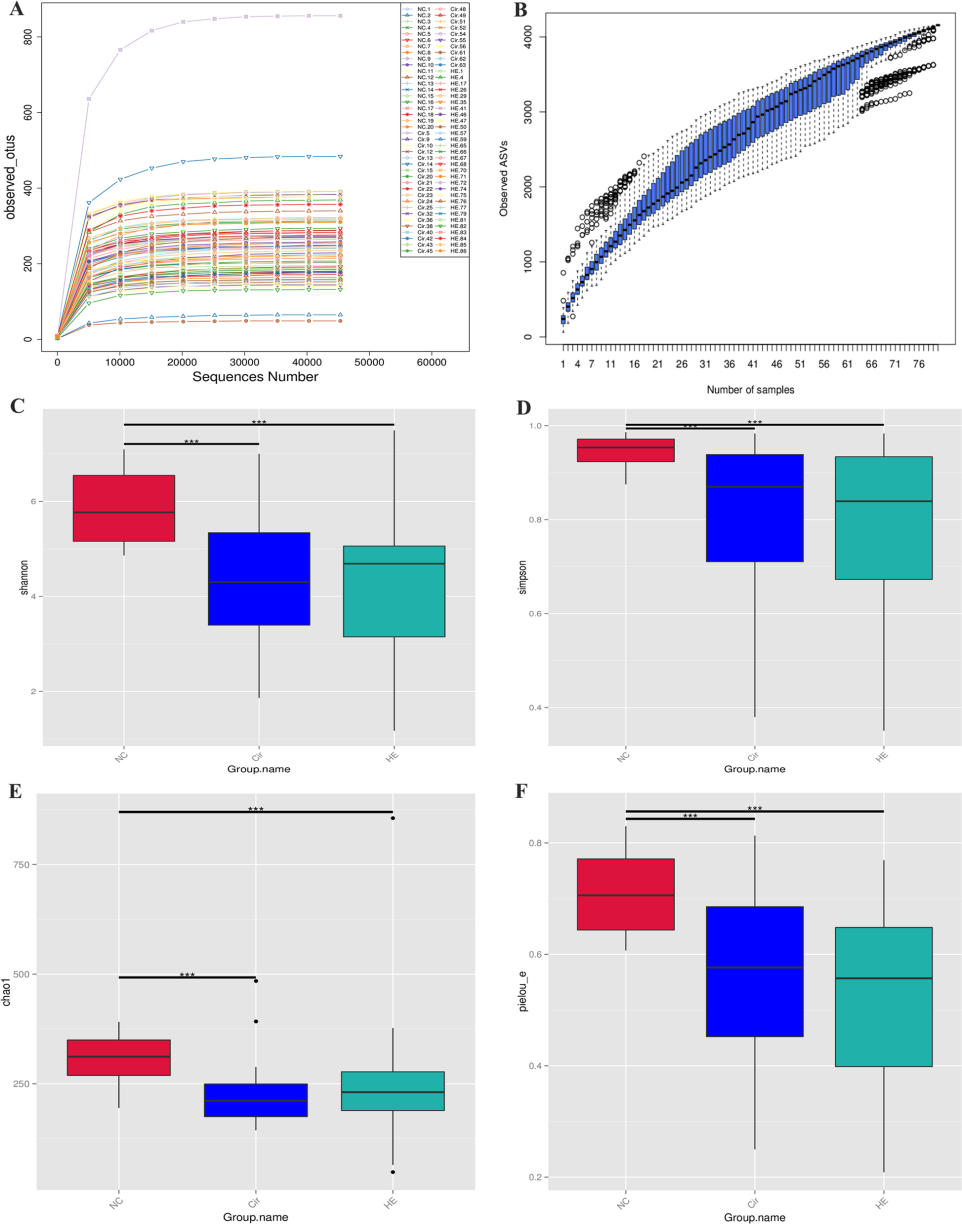
Altered gut microbiota diversity in patients with cirrhosis and HE. **A**, Rarefaction curve in the HE, Cir, and NC groups. **B** Species accumulation boxplots in the HE, Cir, and NC groups. **C**–**F** The α diversity alterations of gut microbiota in the three groups (Shannon, Simpson, Chao1, and Pielou_e index)

**Fig. 2:**
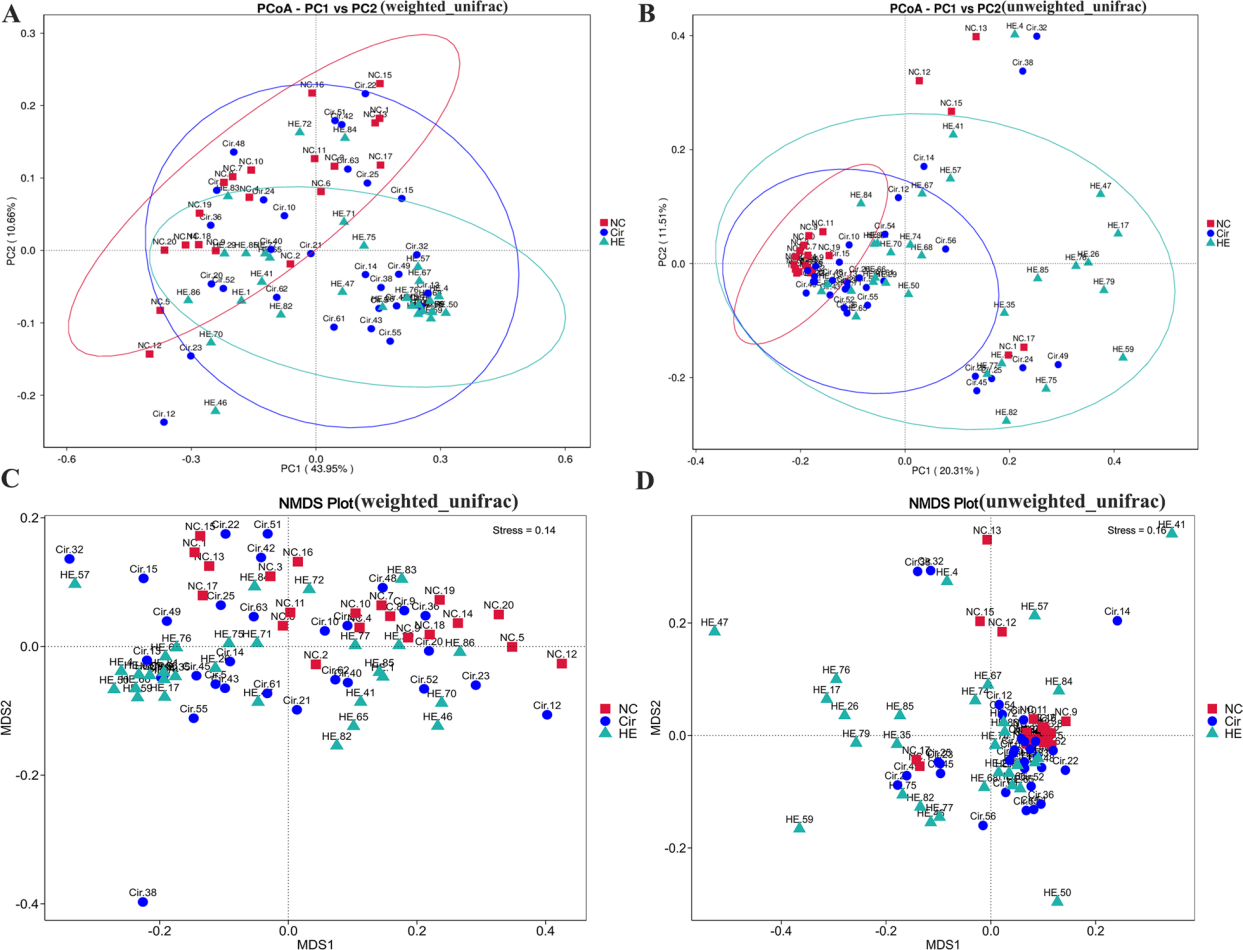
Gut microbiota composition of the three groups. **A**, **B** PCoA analysis using UniFrac distances. **C**, **D** NMDS analysis using Bray‒Curtis distances

The sparse curve and species accumulation boxplots (Fig. 1A, B) indicate that there were reasonable sequencing data and sample sizes. Analysis of α diversity indices (Shannon, Simpson, Chao1, and Pielou_e index) showed that HE and Cir patients had lower species richness and diversity than healthy volunteers (*p* < 0.001) (Fig. 1C–F). It was evident that the gut microbiota compositions of the three groups differed from one another based on the PCoA analysis and the NMDS analysis (Fig. 2A–D).

**Fig. 3:**
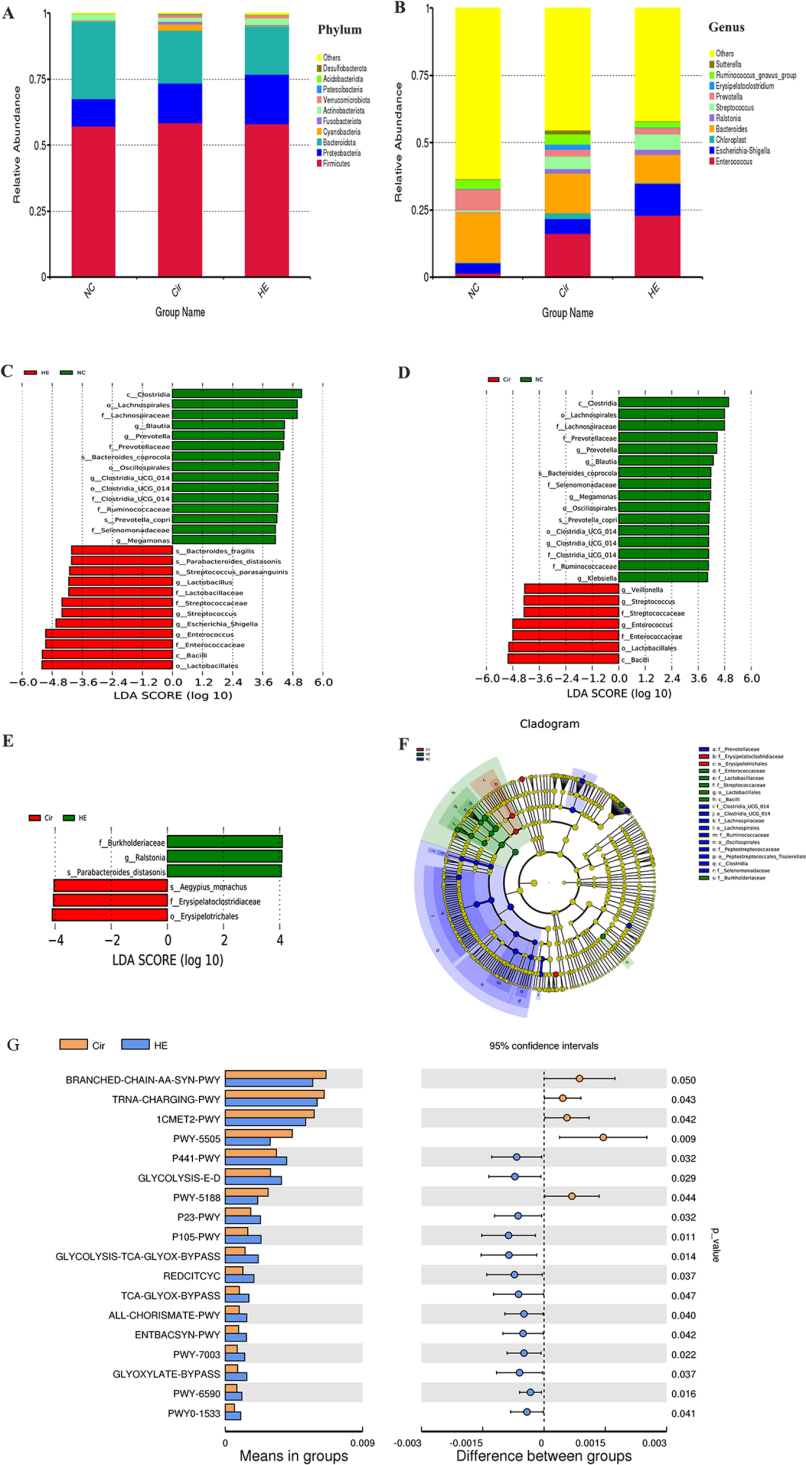
The relative abundances of dominant taxa and the results of LDA effect size analysis of the three groups and function annotations. **A**, **B** Phylum- and genus-level relative abundances of dominant taxa. **C** LDA score for different taxa in the HE group (red) and NC group (green); **D** in the Cir group (red) and NC group (green); **E** in the Cir group (red) and HE group (green). **F** The evolutionary branch diagram of all taxa; circles in blue and green denote the differences between the most abundant classes of microbiota. **G** Results of the functional annotations in the Cir group and HE group based on the MetaCyc database

The prevalent phyla were *Firmicutes*, *Proteobacteria*, *Bacteroidetes*, and *Actinobacteria.* The proportions of bacteria in the HE group were *Firmicutes* (58.08%), *Proteobacteria* (18.83%), *Bacteroidetes* (18.19%), and *Actinobacteria* (2.5%). The Cir group exhibited *Firmicutes* (58.42%), *Proteobacteria* (15.08%), *Bacteroidetes* (20.14%), and *Actinobacteria* (1.6%). The gut microbiota of the NC group comprised *Firmicutes* (57.31%), *Proteobacteria* (10.31%), *Bacteroidetes* (29.34%), and *Actinobacteria* (2.5%). Overall, members of the phylum *Proteobacteria* were more abundant in the HE and Cir groups than in the NC group. However, a lower proportion of *Bacteroidota* was found in the HE and Cir groups than in the NC group (Fig. 3A). The proportions of the most dominant genera in the HE group were *Bacteroides* (10.59%), *Enterococcus* (22.98%), *Prevotella* (2.21%), *Escherichia-Shigella* (11.9%), *Ruminococcus_gnavus* (2.13%), and *Streptococcus* (5.9%). The Cir group exhibited *Bacteroides* (14.80%), *Enterococcus* (16.36%), *Prevotella* (2.55%), *Escherichia-Shigella* (2.55%), *Ruminococcus_gnavus* (5.35%), and *Streptococcus* (3.82%). The NC group exhibited *Bacteroides* (18.61%), *Enterococcus* (1.42%), *Prevotella* (7.82%), *Escherichia-Shigella* (3.95%), *Ruminococcus_gnavus* (3.37%), and *Streptococcus* (0. 63%). The HE group had higher proportions of *Enterococcus*, *Escherichia-Shigella*, and *Streptococcus* than the Cir group. The Cir group had higher abundances of *Enterococcus*, *Escherichia coli*, and *Streptococcus* than the NC group. A lower proportion of *Bacteroides* was found in the Cir group than in the HE group, but a higher proportion was found in the NC group. There was a lower proportion of *Prevotella* in the HE and Cir groups than in the NC group (Fig. 3A). At the family level, the relative abundance of *Lachnospiraceae* and *Ruminococcaceae* was lower in the HE group, whereas the relative abundance of *Enterobacteriaceae* was higher (Additional file 2: Fig. S1C). At the class, order, and species levels, there was an altered relative abundance of gut microbes (Additional file 2: Fig. S1A, B, D).

The results of the LEfSe analysis revealed that each group had a unique gut microbiota structure. There were 27 taxa (containing five grading levels) shared between the HE and NC groups (Fig. 3C), 23 taxa (including five grading levels) shared between the Cir and NC groups (Fig. 3D), and 6 taxa (including four grading levels) shared between the HE group and Cir group with linear discriminant analysis (LDA) values > 4 (Fig. 3E). The evolutionary branch diagram of all taxa is shown in Fig. 3F. The relative abundances of *Lactobacillus*, *Streptococcus*, *Escherichia_Shigella*, and *Enterococcus* in the HE group were considerably lower than those in the NC group. The relative abundance of *Veillonella*, *Streptococcus*, and *Enterococcus* was considerably lower in the Cir group than in the NC group. The HE group showed a much higher relative abundance of *Ralstonia* than the Cir group. At the species level, *Bacteroides fragilis*, *Parabacteroides distaspinus*, and *Streptococcus parasangunis* were significantly more abundant in the NC group than in the HE group. In contrast to *Aegypius monachus*, *Parabacteroides distaspnis* was substantially more abundant in the HE group than in the Cir group.

The analysis of the functional annotations in the MetaCyc database revealed 18 pathways with different activity levels between the HE and Cir groups (Fig. 3G). Among these pathways, 13 were activated, while 5 were inhibited. The Cir group showed activation of different pathways than the NC group (Additional file 2: Fig. S2). The differences in the pathways between the HE and NC groups are shown in Additional file 2: Fig. S3. The changes in these pathways may have caused the changes in the metabolomes. In the gut microbiome, these pathways may be dysregulated or activated, which can influence the metabolome.

### Serum metabolite profiling in cirrhosis and HE patients

#### SCFAs

**Fig. 4:**
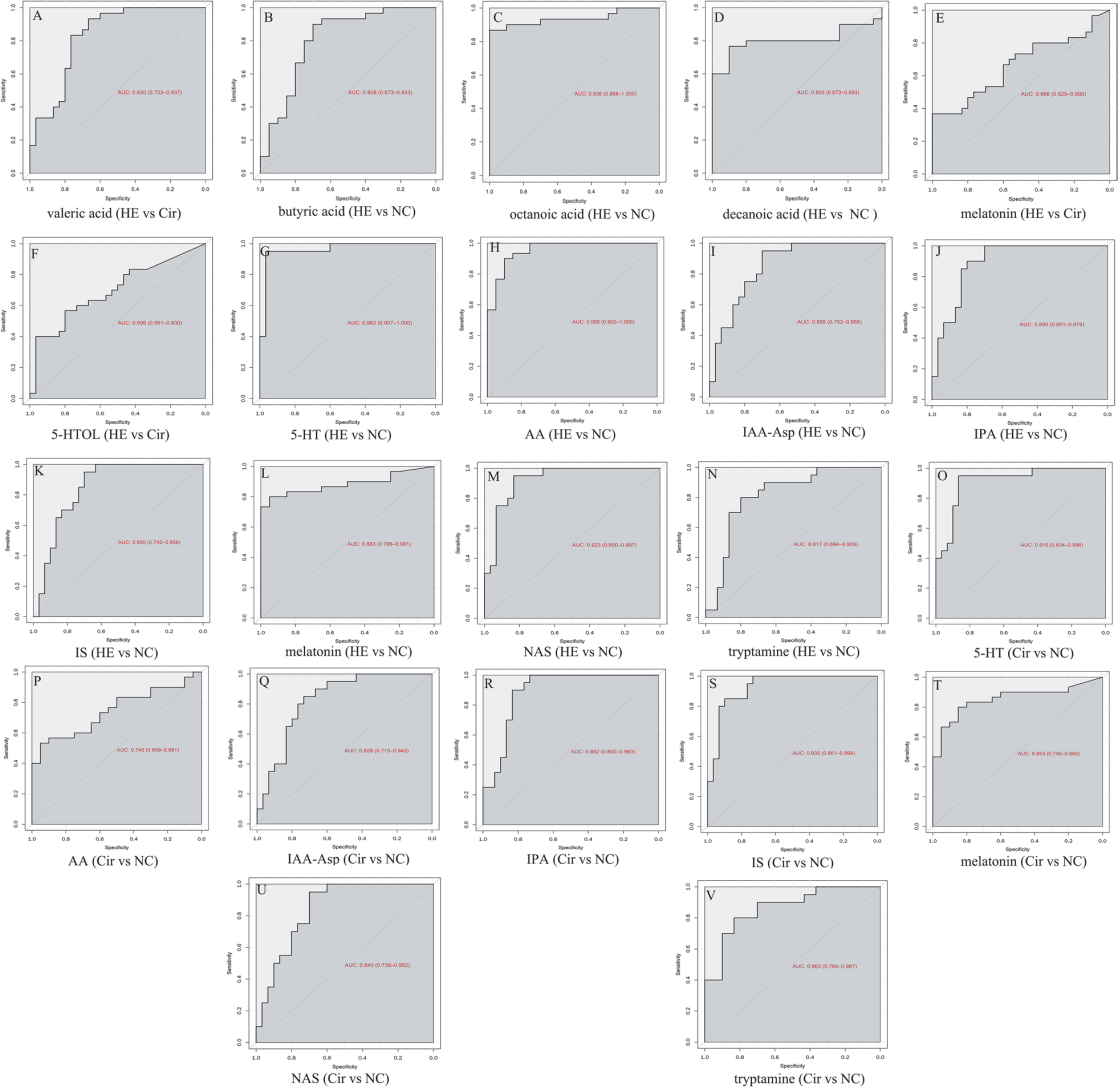
The AUC values achieved using the levels of serum SCFAs (**A**–**D**) and tryptophan metabolites (**E**–**V**)

The concentrations of 11 SCFAs in the HE, Cir, and NC groups are summarized in Additional file 1: Table S1. The HE group had higher concentrations of butyric acid, valeric acid, and hexanoic acid than the Cir group, but the HE group did not have lower concentrations of SCFAs. The concentrations of propionic acid, isobutyric acid, butyric acid, valeric acid, hexanoic acid, octanoic acid, and decanoic acid were higher in the HE group than in the NC group; moreover, the HE group did not have lower concentrations of SCFAs. The Cir group had a higher concentration of propionic acid than the NC group, while the Cir group did not have a lower concentration of SCFAs. Using VIP > 1.0 and FC > 2.0 or < 0.5 as criteria, we identified SCFAs with significant differences in their levels. There was a significant difference in the levels of valeric acid between the HE and Cir groups. A significant difference was found in the butyric acid, octanoic acid, and decanoic acid concentrations between the HE and NC groups. The metabolites detected in the Cir group were identical to those detected in the NC group. Metabolites were evaluated as diagnostic and prognostic markers by plotting receiver operating characteristic (ROC) curves and calculating the area under the curve (AUC). The AUC value achieved during the analysis of valeric acid levels to distinguish the HE group and the Cir group was 0.830. The AUC values achieved using the levels of butyric acid, octanoic acid, and decanoic acid to distinguish the HE group and the NC group were 0.808, 0.938, and 0.803, respectively (Fig. 4A–D).

#### Tryptophans

The serum concentrations of 28 tryptophan metabolites in the three groups are summarized in Additional file 1: Table S2. There were higher concentrations of 6 tryptophans (3-hydroxykynurenine (3-HK), 5-hydroxytryptophol (5-HTOL), 5-methoxy-3-indoleacetic (5-Me-IAA), indolelactic acid (ILA), kynurenine (KYN), and melatonin) and lower concentrations of serotonin (5-HT) in the HE group than in the Cir group. A higher concentration of 12 tryptophans (3-hydroxyanthranilic acid (3-HAA), 3-HK, 5-HTOL, 5-Me-IAA, anthranilic acid (AA), indole-3-acetyl-aspartate (IAA-Asp), indole-3-acetonitrile (IAN), indole ethanol/tryptophol (IE), ILA, indican, KYN, and melatonin) and a lower concentration of 8 tryptophans (5-HT, IPA, indoxylsulfate (IS), *N*-acetyl-5-hydroxytryptamine (NAS), skatole, l-tryptophan (Trp), tryptamine, and xanthurenic acid (Xa)) were found in the HE group than in the NC group. Six tryptophans (3-HAA, 3-HK, AA, IAN, melatonin, and nicotinic acid) had higher levels, whereas 11 tryptophans (5-HT, l-5-hydroxytryptophan (5-HTP), IAA, IAA-Asp, indole-3-carboxaldehyde (ICA), indican, IPA, IS, NAS, Trp, tryptamine, and Xa) had lower levels in the Cir group than in the NC group. The concentrations of melatonin and 5-HTOL were significantly different between the HE and Cir groups. 5-HT, AA, IAA-Asp, IPA, IS, melatonin, NAS, and tryptamine all had significantly different levels between the HE and NC groups. The concentrations of 8 tryptophans in the Cir group and NC group also differed significantly. The AUC values achieved using the levels of melatonin and 5-HTOL to distinguish the HE group and the Cir group were 0.666 and 0.696, respectively. The AUC values achieved using the levels of 5-HT, AA, IAA-Asp, IPA, IS, melatonin, NAS, and tryptamine to distinguish between the HE group and the NC group were 0.962, 0.955, 0.855, 0.89, 0.85, 0.883, 0.923, and 0.817, respectively. The AUC values achieved when distinguishing between the Cir group and the NC group using 5-HT, AA, IAA-Asp, IPA, IS, melatonin, NAS, and tryptamine levels were 0.915, 0.745, 0.828, 0.892, 0.93, 0.853, 0.845, and 0.863, respectively (Fig. 4E–V).

### Faecal metabolite profiling in cirrhotic and HE patients

#### SCFAs

**Fig. 5:**
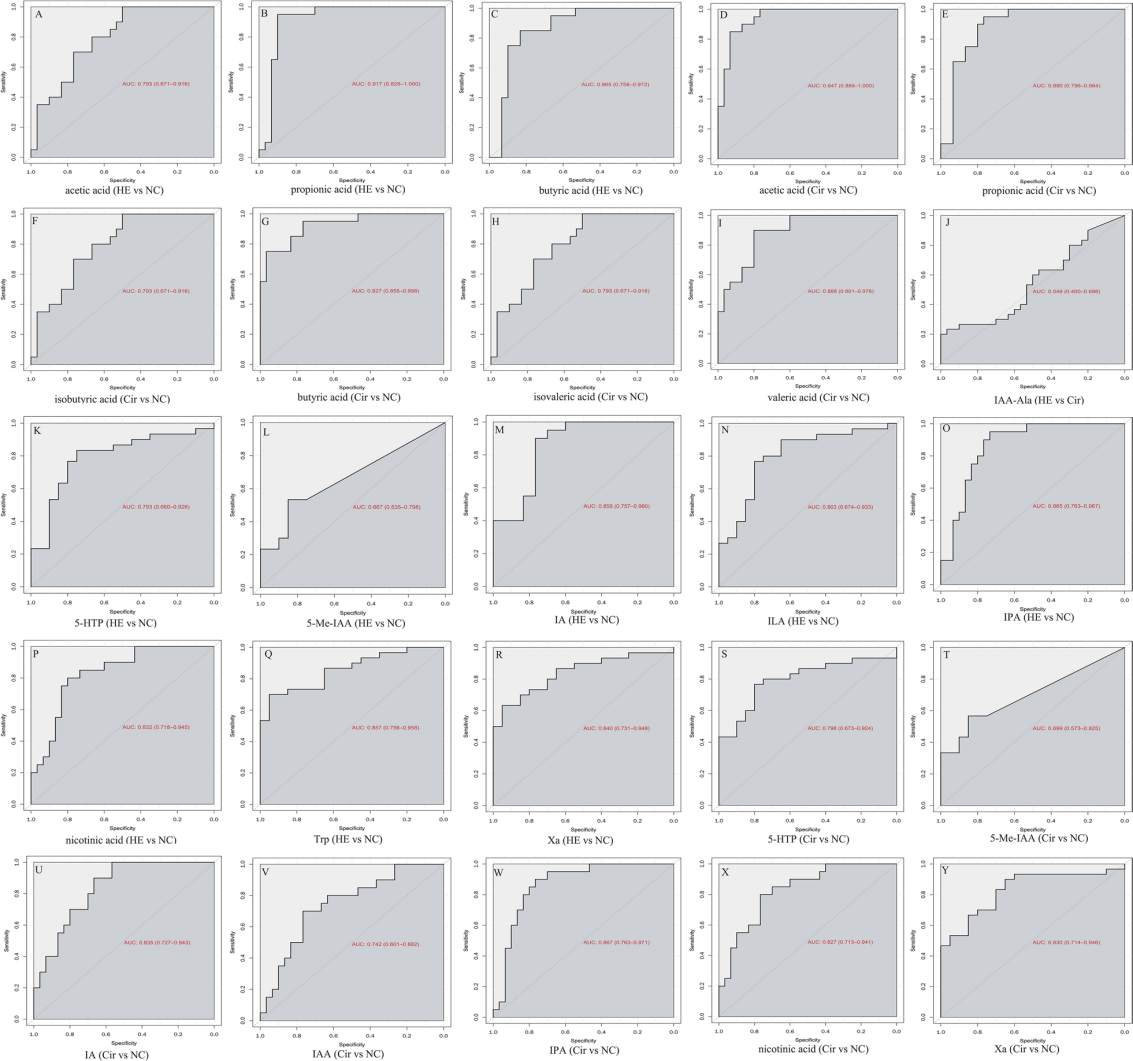
The AUC values achieved using the levels of faecal SCFAs (**A**–**I**) and tryptophan metabolites (**J**–**Y**)

The faecal concentrations of 11 SCFAs in the three groups are summarized in Additional file 1: Table S3. The mean concentrations of SCFAs did not differ between the HE and Cir groups. The mean concentrations of acetic, propionic, butyric, and nonanoic acids were lower in the HE group than in the NC group, although nonanoic acid levels were greater in the HE group. The concentrations of acetic, propionic, isobutyric, butyric, and isovaleric acids were lower in the Cir group than in the NC group. Acetic acid, propionic acid, and butyric acid concentrations were significantly different between the HE and NC groups. The concentrations of acetic acid, propionic acid, isobutyric acid, butyric acid, isovaleric acid, and valeric acid in the Cir group were considerably different from those in the NC group. The AUC values achieved using the levels of acetic acid, propionic acid, and butyric acid to distinguish between the HE group and the NC group were 0.923, 0.917, and 0.865, respectively. The AUC values achieved using the levels of propionic acid, acetic acid, butyric acid, isobutyric acid, valeric acid, and isovaleric acid to distinguish between the Cir group and the NC group were 0.89, 0.947, 0.927, 0.833, 0.883, and 0.793, respectively (Fig. 5A–I).

#### Tryptophans

Table S4 shows the faecal concentrations of 29 tryptophans in the three groups. The HE group had a lower mean concentration of IAA-Ala than the Cir group. None of the tryptophan metabolites had higher levels in the HE group than in the Cir group. The mean concentrations of 5-HTP, 5-Me-IAA, ILA, Trp, and Xa in the HE group were higher than those in the NC group, while the 3-HAA, 5-HIAA, indole acrylic acid (IA), IAA, IPA, and nicotinic acid levels in the HE group were lower than those in the NC group. The concentrations of 5-HTP, 5-Me-IAA, IAA-Ala, Trp, and Xa were found to be higher in the Cir group than in the NC group, whereas the concentrations of IA, IAA, IPA, and nicotinic acid were lower. There were appreciable differences in the levels of IAA-Ala between the HE group and the Cir group. There were significant differences in the levels of 5-HTP, 5-Me-IAA, IA, ILA, IPA, nicotinic acid, Trp, and Xa between the HE group and the NC group. The Cir and NC groups showed significant differences in 5-HTP, 5-Me-IAA, IA, IAA, IPA, nicotinic acid, and Xa levels. The AUC value achieved using the levels of IAA-Ala to distinguish between the HE group and the Cir group was 0.549. The AUC values achieved using the levels of 5-HTP, 5-Me-IAA, IA, ILA, IPA, nicotinic acid, Trp, and Xa to distinguish between the HE group and the NC group were 0.793, 0.667, 0.858, 0.803, 0.865, 0.832, 0.857, and 0.840, respectively. The AUC values achieved using the levels of 5-HTP, 5-Me-IAA, IA, IAA, IPA, nicotinic acid, and Xa to distinguish between the Cir group and the NC group were 0.798, 0.699, 0.835, 0.742, 0.867, 0.827, and 0.830, respectively (Fig. 5J–Y).

#### Correlation between gut microbes and metabolites

**Fig. 6:**
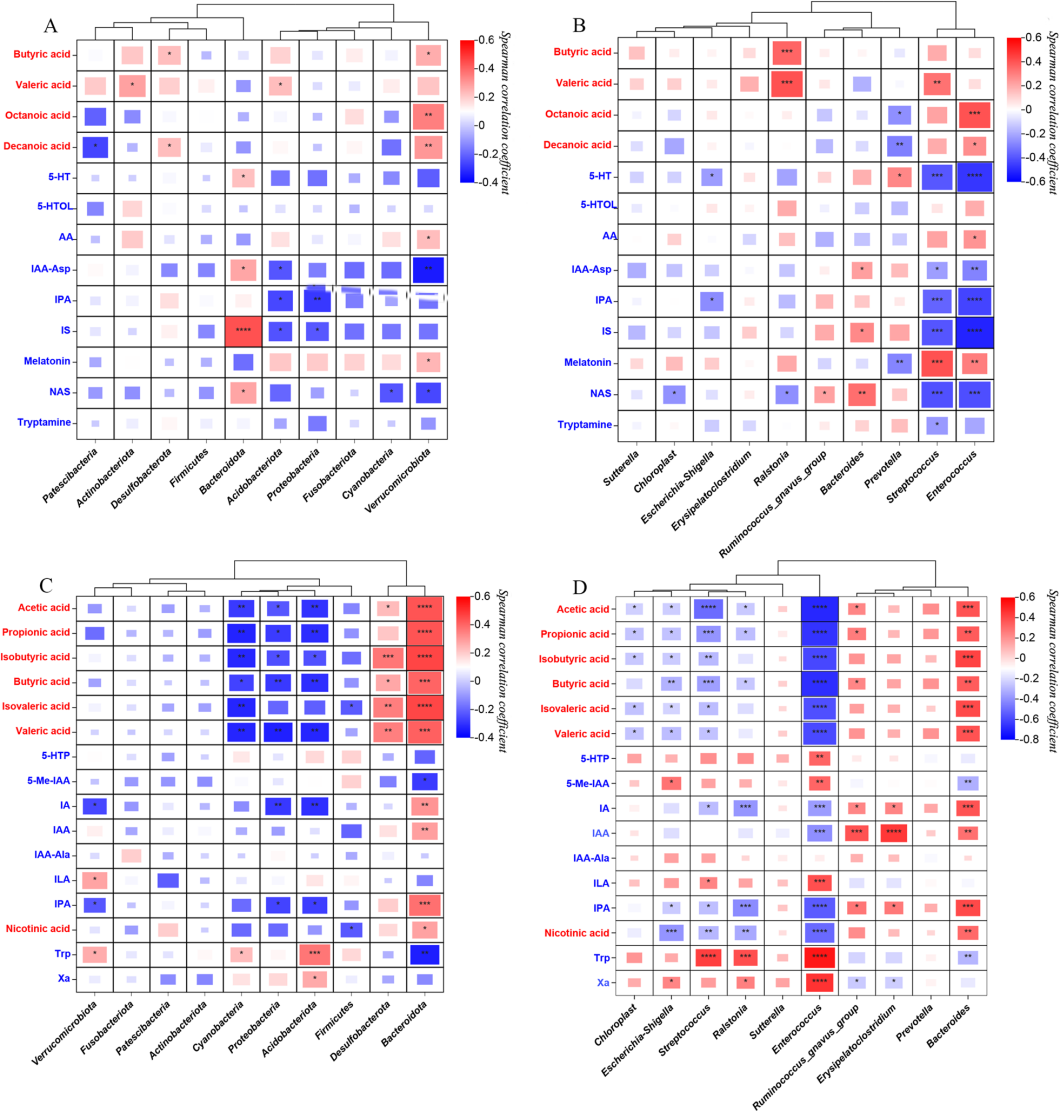
Heatmap of correlation analysis between the levels of metabolites and gut microbes; red represents SCFAs; blue represents tryptophan. **A** Between serum metabolites and gut microbiota at the phylum level. **B** Between serum metabolites and gut microbiota at the genus level. **C** Between faecal metabolites and gut microbiota at the phylum level. **D** Between faecal metabolites and gut microbiota at the genus level

Spearman correlation analysis was conducted to determine the correlation between the levels of differential gut microbes at the phylum and genus levels and the levels of differentially expressed metabolites and to identify relevant microbiota-metabolite combinations based on the criteria |*r*| ≥ 0.5 and *p* < 0.05. No member of the gut microbiota at the phylum level was identified to be closely related to metabolites. At the genus level, *Enterococcus* abundance showed a negative correlation with serum IS levels (*r* = − 0.517, *p* < 0.001). Furthermore, *Enterococcus* abundance was also negatively correlated with acetic acid (*r* = − 0.694, *p* < 0.001), propionic acid (*r* = − 0.66, *p* < 0.001), isobutyric acid (*r* = − 0.606, *p* < 0.001), butyric acid (*r* = − 0.675, *p* < 0.001), isovaleric acid (*r* = − 0.582 *p* < 0.001), valeric acid (*r* = − 0.599, *p* < 0.001), IPA (*r* = − 0.549, *p* < 0.001), and nicotinic acid (*r* = − 0.512, *p* < 0.001) levels in faeces and positively correlated with faecal Trp levels (*r* = 0.538, *p* < 0.001). *Streptococcus* abundance was negatively correlated with faecal acetic acid levels (*r* = − 0.506, *p* < 0.001). Figure 6 displays the heatmaps generated from the correlation analysis, while Table 2 provides a summary of the associated differential gut microbes and metabolites.

**Table 2:** Correlation analysis results of differential bacteria and differential metabolites

	Differential bacteria(genus)	Differential metabolite	r	*p*
Serum	*Enterococcus*	IS	− 0.517	< 0.001
Faecal	*Enterococcus*	Acetic acid	− 0.694	< 0.001
		Propionic acid	− 0.660	< 0.001
		Isobutyric acid	− 0.606	< 0.001
		Butyric acid	− 0.675	< 0.001
		Isovaleric acid	− 0.582	< 0.001
		Valeric acid	− 0.599	< 0.001
		IPA	− 0.549	< 0.001
		Nicotinic acid	− 0.512	< 0.001
		Trp	0.538	< 0.001
	*Streptococcus*	Acetic acid	− 0.506	< 0.001

#### Correlation between serum metabolites and faecal metabolites

**Fig. 7:**
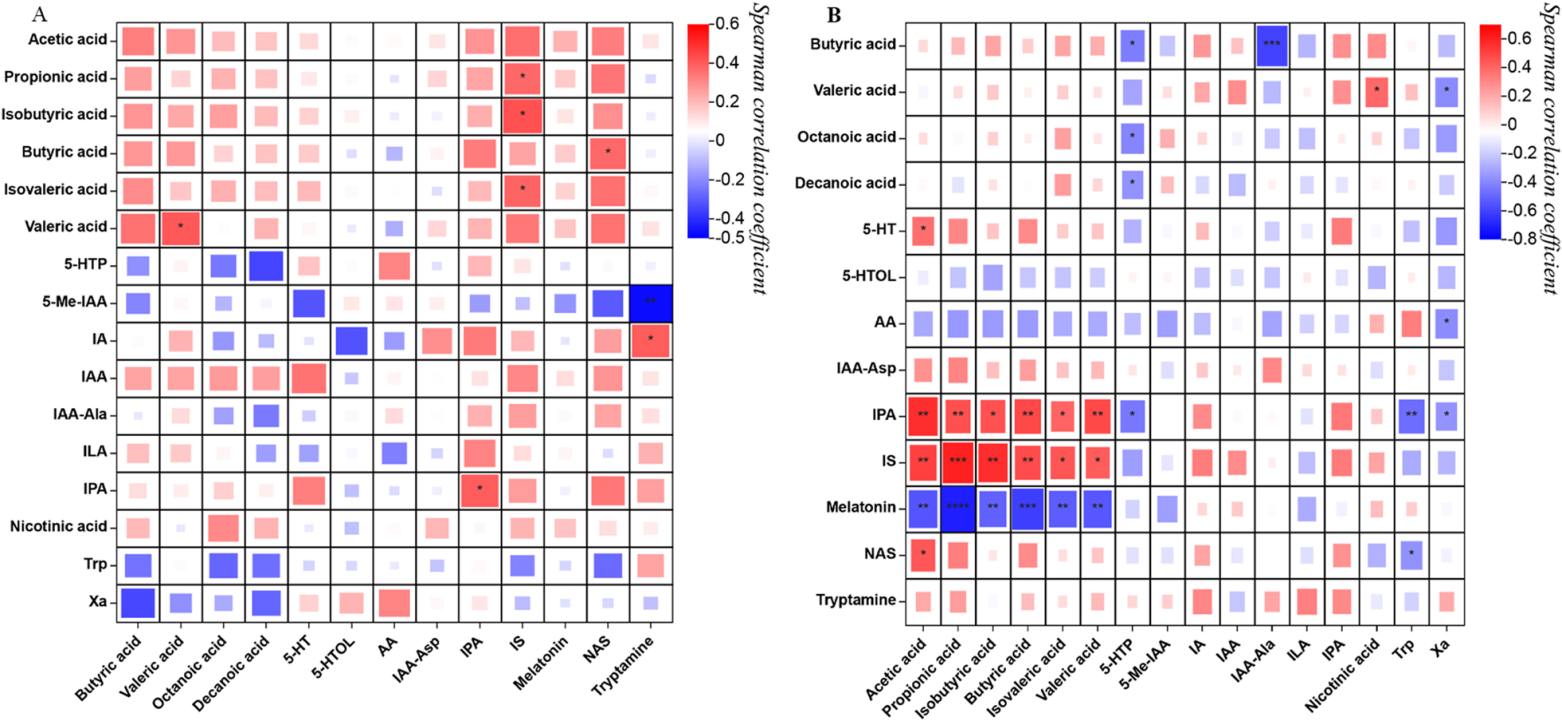
Heatmap of correlation analysis between the levels of serum metabolites and faecal metabolites in the HE group (**A**) and Cir group (**B**). On the left are metabolites in faeces, and the bottom part shows metabolites in serum

The analysis of metabolites in cirrhosis and HE patients revealed the presence of distinct differentially expressed metabolites in both serum and faecal samples. Therefore, we conducted Spearman correlation analyses using the levels of serum and faecal metabolites in different groups (the HE group and the Cir group). The screening criteria were |*r*| ≥ 0.5 and *p* < 0.05. In the HE group, there was no significant correlation between serum and faecal metabolite levels. In the Cir group, serum IPA levels were positively correlated with faecal acetic acid levels (r = 0.564, *p* = 0.001); serum IS levels were positively correlated with acetic acid (r = 0.511, *p* = 0.004), propionic acid (r = 0.607, *p* < 0.001), and isobutyric acid (r = 0.568, *p* = 0.001) levels in faeces; serum melatonin levels were negatively correlated with acetic acid (r = − 0.549, *p* = 0.001), isobutyric acid (r = − 0.514, *p* = 0.003), isovaleric acid (r = − 0.528, *p* = 0.002), and valeric acid (r = − 0.543, *p* = 0.002) levels in faeces; and serum butyric acid levels were negatively correlated with faecal IAA-Ala levels. Figure 7 displays the heatmaps generated from the correlation analysis, while Table 3 provides a summary of the correlated metabolites.

**Table 3:** Correlation analysis results of differential serum and faecal metabolites in the Cir group

Faecal	Serum	r	*p*
Acetic acid	IPA	0.564	0.001
	IS	0.511	0.004
	Melatonin	− 0.549	0.001
Propionic acid	IS	0.607	< 0.001
Isobutyric acid	IS	0.568	0.001
	Melatonin	− 0.514	0.003
Isovaleric acid	Melatonin	− 0.528	0.002
Valeric acid	Melatonin	− 0.543	0.002
IAA-Ala	Butyric acid	− 0.602	< 0.001

#### Correlation between differentially expressed metabolites and clinical parameters

**Fig. 8:**
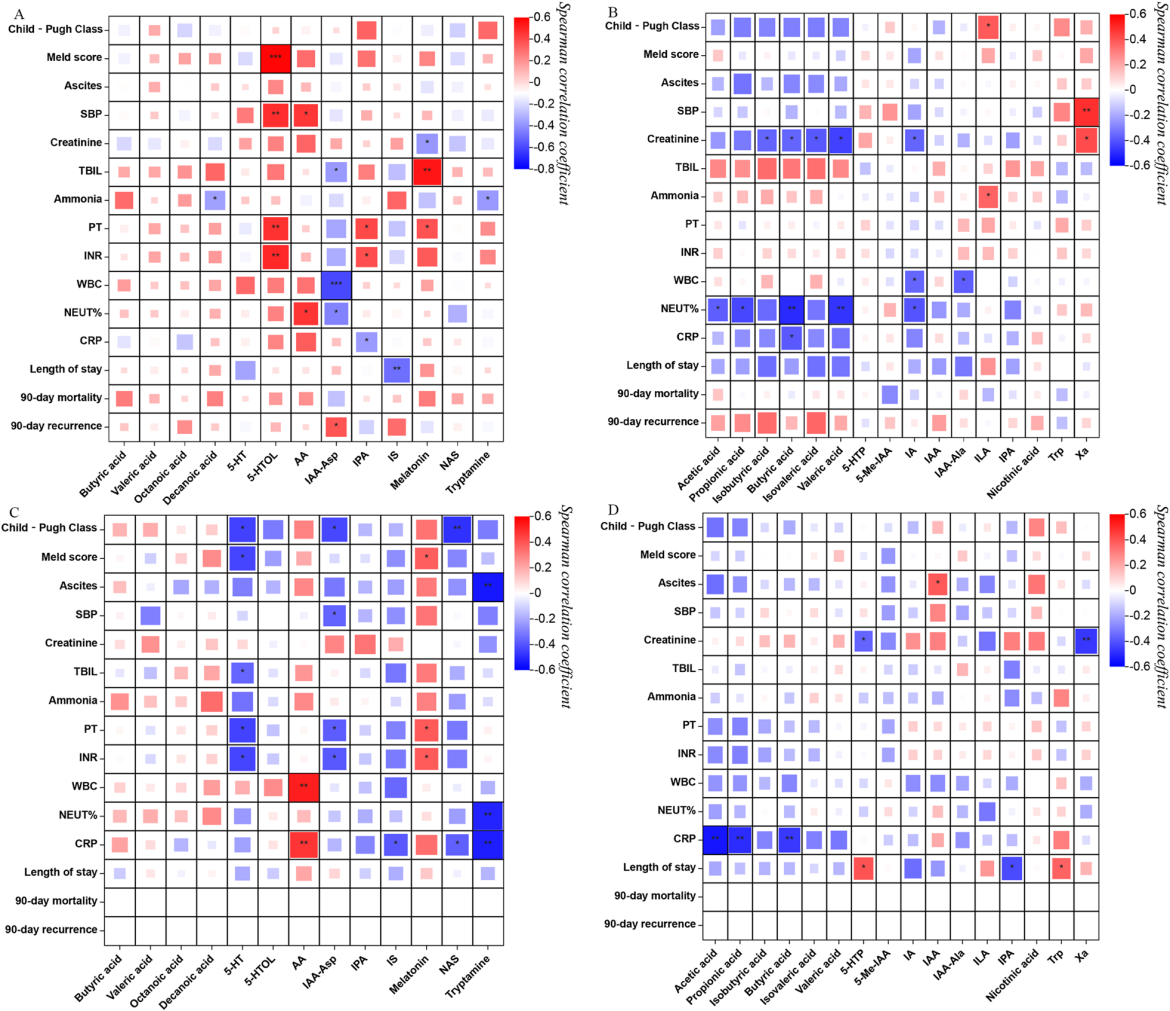
Heatmap of correlation analysis between the levels of metabolites (SCFAs and tryptophan) and clinical parameters. **A** Serum metabolites and clinical parameters of the HE group. **B** Faecal metabolites and the clinical parameters of the HE group. **C** Serum metabolites and the clinical parameters of the Cir group. **D** Faecal metabolites and the clinical parameters of the Cir group

The differentially expressed metabolites were strongly associated with clinical parameters, as revealed by Spearman correlation analysis. In the HE group, serum 5-HTOL levels were positively correlated with Meld score (r = 0.585, *p* < 0.001); serum IAA-Asp levels were positively correlated with WBC (r = 0.542, *p* = 0.002); serum melatonin levels were negatively correlated with TBIL (r = − 0.603, *p* < 0.001); and faecal butyric acid levels were negatively correlated with NEUT% (r = − 0.501, *p* = 0.004). In the Cir group, serum tryptamine levels showed a negative correlation with the presence of ascites (r = − 0.546, *p* = 0.002), NEUT% (r = − 0.518, *p* = 0.003), and CRP levels (r = − 0.531, *p* = 0.003); serum AA levels were positively correlated with WBC (r = 0.528, *p* = 0.003); and a negative correlation was observed between faecal acetic acid and CRP levels (r = − 0.551, *p* = 0.002). Figure 8 presents the heatmaps resulting from the correlation analysis, and Table 4 provides a summary of the metabolites that were correlated with clinical parameters.

**Table 4:** Correlation analysis results of differential metabolites and clinical parameters

		r	*p*		r	*p*
HE group	Serum			Faecal		
Meld score	5-HTOL	0.585	< 0.001			
TBIL	Melatonin	− 0.603	< 0.001			
WBC	IAA-Asp	0.542	0.002			
NEUT%				Butyric acid	− 0.501	0.004
Cir group						
Ascites	Tryptamine	− 0.546	0.002			
WBC	AA	0.528	0.003			
NEUT%	Tryptamine	− 0.518	0.003			
CRP	Tryptamine	− 0.531	0.003	Acetic acid	− 0.551	0.002

## Discussion

**Fig. 9:**
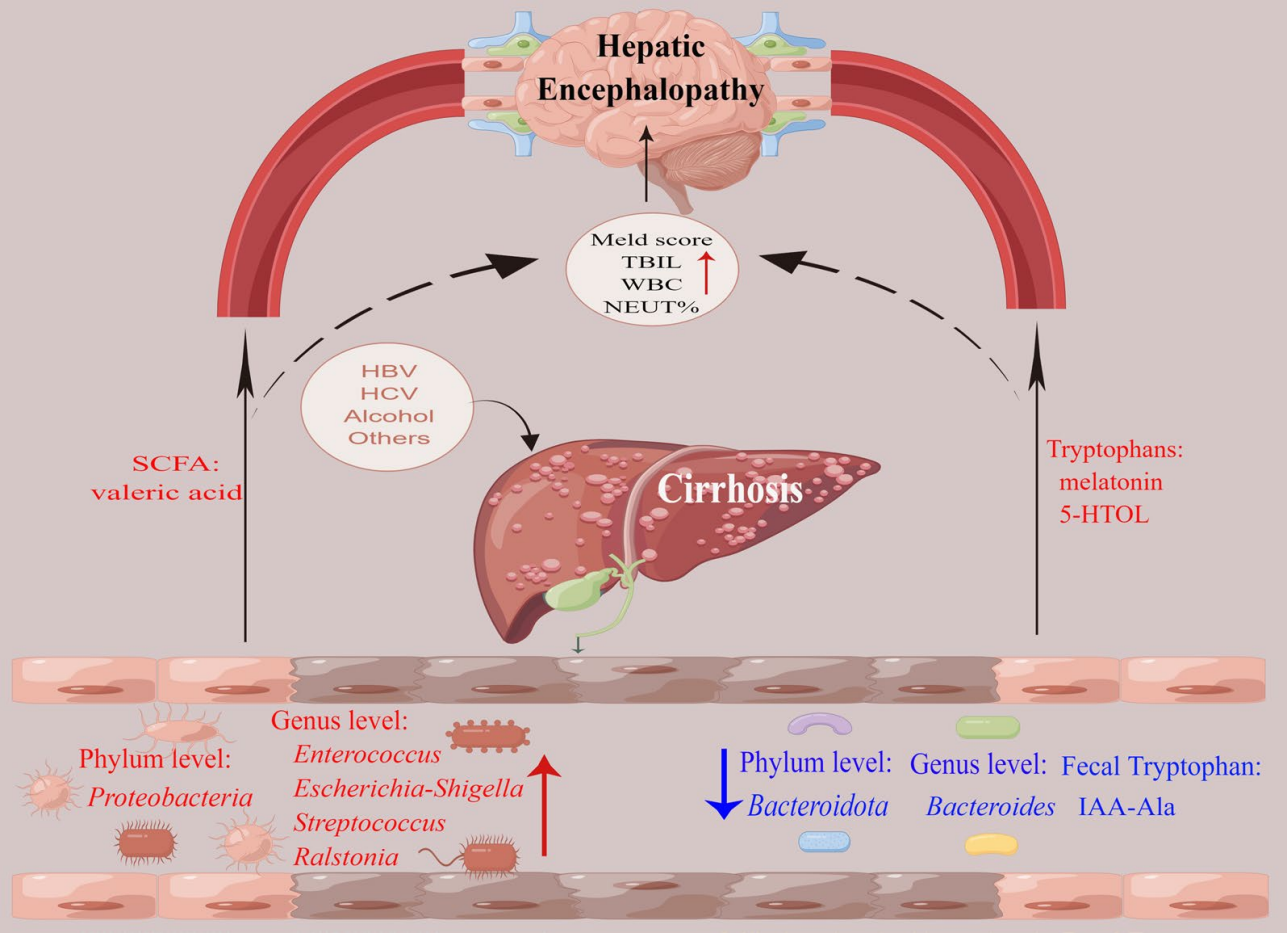
The critical gut microbes, SCFAs, and tryptophan metabolites that may play vital roles in the pathogenesis and progression of cirrhosis to HE. Cirrhosis causes dysbiosis of the gut microbiota and changes in metabolite concentrations in both faeces and serum. These changes result in functional alterations that ultimately contribute to the development of hepatic encephalopathy as the disease progresses. Red represents an increase; blue represents depletion

Recent studies have demonstrated a link between altered gut microbiota structure and liver diseases [33, 34]. It has also been established that microbial-host interactions play roles in the pathogenesis of HE [10]. This study was performed based on previous findings that the gut microbiome is altered in patients with liver diseases, exhibits changes at the phylum or family level, and produces SCFAs and tryptophans with various functions. We identified critical gut microbiota, SCFAs, and tryptophans that have important roles in the pathogenesis and progression of cirrhosis to HE. Our main findings are presented in Fig. 9.

Comparative analysis of clinical parameters and laboratory test indices of the two groups showed that patients with HE had worse liver function. In addition to neuropsychiatric symptoms, HE patients had more severe clinical symptoms (ascites, SBP, and variceal bleeding) than cirrhosis patients. Further investigation revealed that, although the difference between the HE and Cir groups was not statistically significant, the HE group had marginally higher WBC and NEUT% levels. HE has been shown to be associated with systemic inflammation, which is consistent with our findings [35, 36]. HE patients had a slightly worse short-term prognosis than cirrhotic patients in terms of short-term outcomes. Since the p value for the difference in 90-day recurrence was close to 0.05, the limited sample size might have led to the lack of a statistically significant difference. In this study, we found that the richness and diversity of faecal species were lower in patients with HE and cirrhosis than in healthy volunteers, and there were significant alterations in the β-diversity of HE and cirrhosis patients. Our findings suggest a link between alterations in gut microbiota structure and liver diseases. Two prior studies reported comparable findings. One study, comprising 26 MHE patients and 25 cirrhosis patients, revealed that patients had lower diversity than the normal group. Additionally, the β-diversity (Chao1) was not significantly different between MHE patients and cirrhosis patients [37]. Another study revealed that the faecal microbiota differed significantly between patients who had OHE (17 patients) and those who did not have OHE (8 patients) [38]. A more recent study [12], however, showed that patients with a history of OHE (n = 33) had lower diversity than those without a history of OHE (n = 16), which is partially inconsistent with our findings. The inconsistent findings observed among multiple studies could be attributed to inadequate sample sizes. Moreover, our study incorporated patients with both compensated and decompensated cirrhosis in the Cir group.

Specifically, there were discernible variations in the abundance of some taxa in our study. At the phylum level, *Proteobacteria* were more abundant in the Cir group than in the NC group but less abundant in the Cir group than in the HE group. In contrast to the NC group, the HE group had a larger abundance of *Bacteroidota*, which was reduced in the Cir group. Most of the gut’s microbial community comprises *Firmicutes* and *Bacteroidetes*, with lower abundances of *Proteobacteria*, *Actinobacteria*, and *Verrucomicrobia* [39, 40]. *Proteobacteria* have been shown to be associated with metabolic disorders, immune disorders, inflammation, and cancer [41]. *Bacteroidetes* can protect the gut microbiota, and a reduction in the abundance of this microbe has been shown to be associated with obesity [42]. Sung et al. [11] found that the microbiome of acute HE patients had a smaller proportion of the *Bacteroidetes* phylum than that of compensated cirrhosis patients. They also found an increase in the abundance of the *Proteobacteria* phylum, which is similar to what we discovered in our study. A previous study showed that the gut microbiota is a potential marker for diagnosing HE [43], which is also similar to what we discovered in our study. These findings imply that it may be possible to predict the clinical outcomes of patients with HE by analysing the dysbiosis of faecal microbial phyla because it is feasible to assay the gut microbiota at the phylum level in clinical settings [11].

At the genus level, *Enterococcus*, *Escherichia-Shigella*, and *Streptococcus* were more abundant in the HE group than in the Cir group, and these microbes were more abundant in the Cir group than in the NC group. This finding suggests that as the abundance of these three bacteria rises, so does the severity of liver disease. Previous studies have confirmed that *Enterococcus* is associated with poor cognitive function and inflammation in HE [44]. A recent study revealed that *Escherichia-Shigella* abundance was higher in patients with alcoholic cirrhosis than in healthy controls [45]. Qin et al. [7] found that patients with cirrhosis had a higher abundance of *Streptococcus* than healthy controls. In addition, it was discovered that *Streptococcus* is a distinct functional bacterium linked to HE [46]. Furthermore, LEfSe analysis revealed that *Ralstonia* was much more abundant in the HE group than in the Cir group. Notably, *Ralstonia* is widely distributed in the environment, and some species in this genus are associated with plant and crop spoilage [47]. A study by Green et al. revealed that this microbe was also associated with the development of human diseases [48]. They found that the lung function of *Ralstonia*-infected patients with cystic fibrosis was moderately to severely impaired. This is the first study to show that the abundance of *Escherichia-Shigella* and *Ralstonia* is different between patients with HE and cirrhosis. Collectively, these findings indicate that *Enterococcus*, *Escherichia-Shigella*, *Streptococcus*, and *Ralstonia* are potential pathogens involved in HE, and the abundance of these bacteria may influence the prognosis of HE. Furthermore, at the family level, the relative abundance of SCFA-producing families (*Ruminococcaceae* and *Lachnospiraceae*) was decreased. These results are consistent with those from earlier studies [49, 50], which also showed that HE patients had a lower relative abundance of SCFA-producing families. The findings indicate that SCFAs are associated with the occurrence of HE in cirrhosis patients.

The functional annotation analysis revealed that the HE group had 5 inhibited pathways compared to the Cir group. Among these 5 pathways, BRANCHED-CHAIN-AA-SYN-PWY exhibited the highest level of activity. Prior research has demonstrated that the levels of plasma branched-chain amino acids were decreased in individuals with cirrhosis [51] and had an inverse correlation with HE stage [52]. This result was consistent with previous findings. Additionally, P441-PWY, which is associated with N-acetylneuraminic acid, had the highest activity among the 13 activated pathways. We speculate that N-acetylneuraminic acid may contribute to the occurrence of HE in cirrhosis patients. However, no study has reported this relationship, and further investigation is warranted.

We adopted a targeted metabolic approach to measure the levels of SCFAs and tryptophans in the serum and faeces of patients with cirrhosis and HE, in addition to assessing changes in their gut microbiota. This study sought to ascertain whether there were variations in the serum and faecal concentrations of these two types of metabolites between HE and cirrhosis patients and between cirrhosis patients and healthy individuals. Another aim was to clarify whether the alterations in metabolites were the same in serum and faeces. Thus, we attempted to use the analysis of metabolites to establish a connection between the gut microbiota, clinical parameters, and metabolites themselves. In this study, higher concentrations of serum butyric acid, valeric acid, and hexanoic acid were observed in the HE group than in the Cir group, but there were no differences in the levels of these metabolites in faeces. The differences in faecal SCFA levels were inconsistent with those of a recent study [12], which indicated that OHE patients had a lower SCFA content in their faeces. However, the observed variations in serum SCFA levels did not align with the findings of prior research [6] that proposed a correlation between reduced SCFA levels and advanced liver disease in individuals with cirrhosis. As previously reported, the faecal acetic acid concentration reflects its absorption from the colon rather than its production [53]. Therefore, it is possible that SCFAs may be absorbed into the bloodstream more readily due to the reduced intestinal barrier function and increased permeability in HE patients. Propionic acid levels in the serum were higher in the Cir group than in the NC group, but there was no difference in the concentrations of isobutyric acid, acetic acid, isovaleric acid, butyric acid, and valeric acid. The level of propionic acid, however, was lower in faeces, while the levels of the other five SCFAs were not different. The findings highlight the following key points. First, although the same SCFAs were detected in both serum and faeces, the patterns of the differences in their levels were distinct. Second, it appears that SCFAs present in serum, rather than faeces, may be more strongly correlated with HE. Third, the presence of valerate acid in serum may be closely related to the progression of cirrhosis, despite its relatively low levels in faeces.

Regarding tryptophan metabolites, the mean concentrations of serum 3-HK, 5-HTOL, 5-Me-IAA, ILA, KYN, and melatonin were higher in the HE group than in the Cir group, whereas serum 5-HT levels were lower in the HE group. However, the mean concentrations of these 7 tryptophan metabolites were not different in faecal samples collected from these two groups. The HE group had lower faecal IAA-Ala levels than the Cir group; however, the serum levels of IAA-Ala were not different between the two groups. In the Cir group, the level of serum nicotinic acid was higher than that in the NC group; however, it was lower in faecal samples. Serum 5-HTP, Trp, and Xa concentrations were lower in the Cir group, but the levels of these metabolites were higher in faeces. The findings showed that the levels of tryptophan metabolites had distinct patterns of difference in serum and faecal samples. In both serum and faeces, individual tryptophan metabolites showed varying levels.

Subsequently, we analysed the association between these two types of metabolites and the gut microbiota among the three groups, as well as the associations between these two types of metabolites and clinical parameters in two groups (HE and Cir). Correlation analysis implied that *Enterococcus* and *Streptococcus* contribute to the pathogenesis of cirrhosis by regulating partial SCFA and tryptophan synthesis. Furthermore, serum SCFA levels were significantly correlated with tryptophan metabolite levels in faeces but not with faecal SCFA levels. In addition, this showed that serum SCFA levels and tryptophan levels were not correlated with the contents of these metabolites in faeces. Results of the correlation analysis of the levels of metabolites and clinical parameters indicated that the levels of some serum tryptophans were significantly correlated with liver function and systemic inflammation in HE patients, but the levels of SCFAs were not. Conversely, systemic inflammation in patients with HE was found to be correlated with the levels of only one faecal SCFA, specifically butyric acid. In patients with cirrhosis, the levels of some serum tryptophans were significantly correlated with the presence of ascites and systemic inflammation, and the levels of only one faecal SCFA (acetic acid) were correlated with systemic inflammation. However, we did not identify metabolites associated with HE recurrence or mortality.

Considering the substantial influence and crucial role of microbiota-host interactions in HE, it is logical for research in this area to focus on targeting the gut microbiota for HE therapies. Multiple treatments for cirrhosis patients have been studied, including direct approaches that aim to alleviate dysbiosis or decrease the levels of harmful taxa, as well as methods that target microbiota metabolites, such as probiotics and prebiotics [54, 55], faecal microbiota transplantation [56], antibiotics [57], and dietary intervention [58]. In our study, we identified specific microbial genera and SCFA-producing families as well as key metabolites that are linked to the progression of HE in patients with cirrhosis. Our findings may contribute to the treatment of HE with precise and individualized methods.

In our study, there were several potential confounding factors that may have impacted the association between the conditions assessed in this study and outcome variables. These include demographic characteristics, such as sex and BMI; comorbidities, such as diabetes and mental illness; and lifestyle factors, such as diet. We carefully selected our study population based on strict inclusion and exclusion criteria to address these potential confounding factors. As a result, there were no significant differences in sex, BMI, or aetiology among the groups, which ensured that any observed differences in gut microbiota composition and metabolite levels were not confounded by these factors. Strict inclusion and exclusion criteria can effectively eliminate biases introduced by underlying medical conditions. In this study, we opted to maintain the participants’ eating habits instead of altering their diets. This decision implies that it was not entirely possible to eliminate the potential impact of dietary or health-related factors on the identified distinct metabolites. However, the occurrence of HE in cirrhotic patients is unpredictable. In consideration of this, further research adopting a uniform dietary structure for patients should be conducted.

Despite these new findings, this study still has some limitations. First, the sample size in each group was small. The number of SCFAs and tryptophans found to be associated with HE or cirrhosis may increase as the sample size increases. Second, the concentrations of indole, IGA, and IAA-Ala in serum, as well as melatonin and indican in faeces, were too low to be measured quantitatively. Third, we did not distinguish patients with compensated from those with decompensated cirrhosis; thus, it is unclear whether the gut microbiota differs between these two groups. Fourth, our study only focused on SCFAs and tryptophans, and we cannot rule out that other metabolites may also be associated with HE or cirrhosis. Fifth, our study was conducted in a single hospital, which may limit the generalizability of the findings to other populations and settings. Studies including larger sample sizes and more diverse populations are needed to confirm our findings. Finally, MHE patients and patients with other liver diseases were not included; hence, we were unable to explore the association of SCFAs and tryptophans with the severity of HE.

## Conclusion

Species richness and diversity were reduced, and β-diversity was altered in patients with HE and cirrhosis. Genera of *Enterococcus*, *Shigella*, *Streptococcus* and *Ralstonia* and SCFA-producing families (*Ruminococcaceae* and *Lachnospiraceae*) may be associated with the progression of HE in cirrhosis patients. The concentrations of valeric acid, melatonin, and 5-HTOL in serum and faecal IAA-Ala were different between HE patients and cirrhosis patients. Similarly, the concentrations of 5-HT, AA, IAA-Asp, IPA, IS, melatonin, NAS, and tryptamine in serum and acetic acid, propionic acid, isobutyric acid, butyric acid, isovaleric acid, valeric acid, 5-HTP, 5-Me-IAA, IA, IAA, IPA, nicotinic acid, and Xa in faeces were different between cirrhosis patients and healthy volunteers. *Enterococcus* abundance showed a negative association with serum IS levels and acetic acid, propionic acid, isobutyric acid, butyric acid, isovaleric acid, valeric acid, IPA, and nicotinic acid levels in faeces. *Streptococcus* abundance was negatively correlated with faecal acetic acid levels. Serum SCFA levels were significantly correlated with the levels of tryptophans in faeces but not with faecal SCFA levels. Moreover, the levels of some serum tryptophans but not SCFAs were correlated with liver function and systemic inflammation in HE patients. Faecal butyric acid levels were correlated with systemic inflammation in HE patients. The levels of some serum tryptophans were correlated with ascites and systemic inflammation, whereas faecal acetic acid levels were correlated with systemic inflammation in patients with cirrhosis. In addition to gut microbes, we identified key metabolites associated with HE and cirrhosis that are likely to be potential treatments for these conditions.

## Supplementary Information

**Additional file 1: Table S1** The serum concentrations of 11 SCFAs in the three groups. **Table S2** The serum concentrations of 28 tryptophan metabolites in the three groups. **Table S3**: The faecal concentrations of 11 SCFAs in the threegroups. Table S4 The faecal concentrations of 29 tryptophan metabolites in the three groups.

**Additional file 2: Figure S1** The relative abundances of dominant taxa at class level (A), order level (B), family level (C), and species (D). **Figure S2** Analysis result of the function annotationsin Cir group and NC group based on the MetaCyc database. **Figure S3** Analysis result of the function annotationsin HE group and NC group based on the MetaCyc database.

**Additional file 3.** The methods for quantifying the concentrations of SCFAs and tryptophan metabolites.

## Data Availability

The data supporting this study’s findings are available in this article’s supplementary material.
