# Supplementary material for: Integrative analysis of the gut microbiota and faecal and serum short-chain fatty acids and tryptophan metabolites in patients with cirrhosis and hepatic encephalopathy
Source: J Transl Med. 2023 Jun 17;21:395. doi: 10.1186/s12967-023-04262-9 (PMC10276405; doi:10.1186/s12967-023-04262-9)
Supplement: Supplementary file 1 — Additional file 1: Table S1 The serum concentrations of 11 SCFAs in the three groups. Table S2 The serum concentrations of 28 tryptophan metabolites in the three groups. Table S3: The faecal concentrations of 11 SCFAs in the threegroups. Table S4 The faecal concentrations of 29 tryptophan metabolites in the three groups. [file 12967_2023_4262_MOESM1_ESM.docx]

***Additional file 1 (Table)***

Table S1 The serum concentrations of 11 SCFAs in the three groups.

Table S1-1: Comparison of mean serum concentrations of 11 SCFAs in the three groups.

| **Compound name** | **Mean HE** | **Mean Cir** | **Mean NC** | **ANOVA P-VALUE** |
| --- | --- | --- | --- | --- |
| Acetic acid | 2.965217456 | 1.220330002 | 1.183881174 | 0.31883493 |
| Propionic acid | 0.352657327 | 0.250989036 | 0.165064772 | 0.009827567 |
| Isobutyric acid | 0.154235553 | 0.139524828 | 0.129726789 | 0.019683961 |
| Butyric acid | 0.160506064 | 0.09237351 | 0.077464333 | 0.009113121 |
| Isovaleric acid | 0.045425459 | 0.030602821 | 0.032392969 | 0.248060366 |
| Valeric acid | 0.037373239 | 0.012669189 | 0.012945965 | 0.060158795 |
| Hexanoic acid | 0.105881287 | 0.080762017 | 0.073557011 | 0.006315623 |
| Heptanoic acid | 0.027502895 | 0.027444633 | 0.029947559 | 0.541634857 |
| Octanoic acid | 0.443643454 | 1.137428753 | 0.14200281 | 0.448393703 |
| Nonanoic acid | 1.031181617 | 1.053034739 | 1.195668451 | 0.086762708 |
| Decanoic acid | 0.443962666 | 1.142786671 | 0.127549023 | 0.377604487 |

Table S1-2 Comparison of mean serum concentrations of 11 SCFAs between HE and Cir groups.

| **Compound name** | **Mean HE** | **Mean Cir** | **VIP** | **P-VALUE** | **FOLD CHANGE** |
| --- | --- | --- | --- | --- | --- |
| Acetic acid | 2.965217456 | 1.220330002 | 1.134536605 | 0.250081322 | 2.429848854 |
| Propionic acid | 0.352657327 | 0.250989036 | 0.556016379 | 0.199108446 | 1.405070649 |
| Isobutyric acid | 0.154235553 | 0.139524828 | 0.754281456 | 0.171291408 | 1.105434463 |
| Butyric acid | 0.160506064 | 0.09237351 | 1.365952032 | 0.024512481 | 1.737576753 |
| Isovaleric acid | 0.045425459 | 0.030602821 | 0.759223732 | 0.102568529 | 1.484355268 |
| Valeric acid | 0.037373239 | 0.012669189 | 1.736102844 | 0.019888322 | 2.949931355 |
| Hexanoic acid | 0.105881287 | 0.080762017 | 1.438884331 | 0.023299316 | 1.311028258 |
| Heptanoic acid | 0.027502895 | 0.027444633 | 0.059617065 | 0.980274992 | 1.002122894 |
| Octanoic acid | 0.443643454 | 1.137428753 | 0.180627143 | 0.426017781 | 0.390040653 |
| Nonanoic acid | 1.031181617 | 1.053034739 | 1.124232479 | 0.560141575 | 0.979247482 |
| Decanoic acid | 0.443962666 | 1.142786671 | 0.08925303 | 0.384429457 | 0.388491288 |

Note: VIP, Variable Importance in Projection; red means increase, green means decrease.

Table S1-3 Comparison of mean serum concentrations of 11 SCFAs between HE and NC groups.

| **Compound name** | **Mean HE** | **Mean NC** | **VIP** | **P-VALUE** | **FOLD CHANGE** |
| --- | --- | --- | --- | --- | --- |
| Acetic acid | 2.965217456 | 1.183881174 | 0.553202117 | 0.241231118 | 2.504658002 |
| Propionic acid | 0.352657327 | 0.165064772 | 0.738646778 | 0.012364013 | 2.136478441 |
| Isobutyric acid | 0.154235553 | 0.129726789 | 0.708983556 | 0.013764402 | 1.188926006 |
| Butyric acid | 0.160506064 | 0.077464333 | 1.125332673 | 0.010990223 | 2.071999558 |
| Isovaleric acid | 0.045425459 | 0.032392969 | 0.283643697 | 0.138820161 | 1.402324658 |
| Valeric acid | 0.037373239 | 0.012945965 | 0.826976595 | 0.021286155 | 2.886863857 |
| Hexanoic acid | 0.105881287 | 0.073557011 | 1.132717111 | 0.002780427 | 1.439445202 |
| Heptanoic acid | 0.027502895 | 0.029947559 | 0.540104396 | 0.340560685 | 0.918368515 |
| Octanoic acid | 0.443643454 | 0.14200281 | 1.723896784 | 0.000153036 | 3.124187841 |
| Nonanoic acid | 1.031181617 | 1.195668451 | 0.677650877 | 0.14677486 | 0.862431066 |
| Decanoic acid | 0.443962666 | 0.127549023 | 1.615302252 | 0.011881917 | 3.480721801 |

Table S1-4 Comparison of mean serum concentrations of 11 SCFAs between Cir and NC groups.

| **Compound name** | **Mean Cir** | | **Mean NC** | **VIP** | **P-VALUE** | **FOLD CHANGE** |
| --- | --- | --- | --- | --- | --- | --- |
| Acetic acid | 1.220330002 | 1.18388117 | | 0.215850622 | 0.735884379 | 1.030787573 |
| Propionic acid | 0.250989036 | 0.16506477 | | 0.60952818 | 0.041960983 | 1.520548766 |
| Isobutyric acid | 0.139524828 | 0.12972679 | | 0.796283497 | 0.106772364 | 1.075528261 |
| Butyric acid | 0.09237351 | 0.07746433 | | 0.848845364 | 0.366141303 | 1.192465055 |
| Isovaleric acid | 0.030602821 | 0.03239297 | | 0.53240974 | 0.781480409 | 0.944736539 |
| Valeric acid | 0.012669189 | 0.01294597 | | 0.232443041 | 0.886509139 | 0.97862069 |
| Hexanoic acid | 0.080762017 | 0.07355701 | | 0.397854983 | 0.170697371 | 1.097951317 |
| Heptanoic acid | 0.027444633 | 0.02994756 | | 0.854722125 | 0.273257876 | 0.916423046 |
| Octanoic acid | 1.137428753 | 0.14200281 | | 1.89953932 | 0.254726086 | 8.009903115 |
| Nonanoic acid | 1.053034739 | 1.19566845 | | 0.948849653 | 0.207954292 | 0.880707974 |
| Decanoic acid | 1.142786671 | 0.12754902 | | 1.868792219 | 0.205020011 | 8.95958779 |

Table S2 The serum concentrations of 28 tryptophan metabolites in the three groups.

Table S2-1: Comparison of mean serum concentrations of 28 tryptophans in the three groups.

| **Compound name** | **Mean HE** | **Mean Cir** | **Mean NC** | **ANOVA P-VALUE** |
| --- | --- | --- | --- | --- |
| 3-HAA | 43.30562567 | 33.86680581 | 22.07486559 | 0.030908568 |
| 3-HK | 108.9492639 | 49.67311544 | 25.95309726 | 0.000117513 |
| 5-HIAA | 143.0998197 | 56.38231512 | 63.06782174 | 0.125372994 |
| 5-HT | 106.3271894 | 181.0425459 | 576.487183 | 1.10068E-09 |
| 5-HTOL | 1.597036705 | 0.611750862 | 0.388687879 | 0.012746445 |
| 5-HTP | 9.641881481 | 8.317155414 | 10.75648836 | 0.093088107 |
| 5-Me-IAA | 0.575917982 | 0.326361181 | 0.312576983 | 0.011648953 |
| AA | 25.73229197 | 16.32642128 | 7.051492211 | 5.30928E-05 |
| IA | 1.677324189 | 0.954832049 | 7.356062995 | 0.109748334 |
| IAA | 1677.185629 | 1348.875703 | 2027.012495 | 0.027046606 |
| IAA-Asp | 0.810406613 | 0.966186302 | 2.503771371 | 0.004750892 |
| IAM | 0.69545239 | 0.558777756 | 0.708962694 | 0.71191165 |
| IAN | 4.733858916 | 3.149119297 | 2.113546604 | 0.005758484 |
| ICA | 74.56128894 | 64.93884112 | 95.29989697 | 0.016706906 |
| IE | 2.052289671 | 0.504605769 | 0.231481645 | 0.042184553 |
| ILA | 1935.7569 | 1084.504961 | 951.6583913 | 0.017044007 |
| Indican | 132.9708177 | 163.6299763 | 222.0054694 | 2.16204E-06 |
| IPA | 412.1209144 | 396.65219 | 2704.21881 | 0.032098122 |
| IS | 1811.642717 | 766.1269376 | 4004.423995 | 0.001329634 |
| KYN | 2980.040274 | 2207.385322 | 1898.551844 | 0.001906437 |
| KYNA | 226.2638217 | 59.24751944 | 45.68366565 | 0.07618947 |
| Melatonin | 0.534356969 | 0.251453085 | 0.043426903 | 1.80247E-08 |
| Nicotinic acid | 2.355298121 | 3.494814294 | 1.981427648 | 0.013192085 |
| NAS | 0.098590597 | 0.152868919 | 0.309939586 | 6.10481E-08 |
| Skatole | 12.33555212 | 14.82829054 | 16.9930479 | 0.022687138 |
| Trp | 50369.72727 | 54834.04073 | 66415.04192 | 0.010208919 |
| Tryptamine | 0.221192184 | 0.183222803 | 0.469252178 | 1.10874E-05 |
| Xa | 100.9375748 | 101.5191146 | 124.1776562 | 0.000485775 |

Table S2-2 Comparison of mean serum concentrations of 28 tryptophans between HE and Cir groups.

| **Compound name** | **Mean HE** | **Mean Cir** | **VIP** | **P-VALUE** | **FOLD CHANGE** |
| --- | --- | --- | --- | --- | --- |
| 3-HAA | 43.30562567 | 33.86680581 | 0.757940384 | 0.239389603 | 1.278704166 |
| 3-HK | 108.9492639 | 49.67311544 | 1.603212286 | 0.016084116 | 2.193324557 |
| 5-HIAA | 143.0998197 | 56.38231512 | 1.436144589 | 0.108759742 | 2.538026674 |
| 5-HT | 106.3271894 | 181.0425459 | 1.431391811 | 0.044354809 | 0.587304983 |
| 5-HTOL | 1.597036705 | 0.611750862 | 1.096353107 | 0.037626473 | 2.610599842 |
| 5-HTP | 9.641881481 | 8.317155414 | 0.254784342 | 0.284853495 | 1.159276339 |
| 5-Me-IAA | 0.575917982 | 0.326361181 | 1.73492686 | 0.006257438 | 1.764664477 |
| AA | 25.73229197 | 16.32642128 | 1.460243774 | 0.089560829 | 1.576113437 |
| IA | 1.677324189 | 0.954832049 | 0.009002988 | 0.409746647 | 1.756669344 |
| IAA | 1677.185629 | 1348.875703 | 0.627652732 | 0.144170624 | 1.243395241 |
| IAA-Asp | 0.810406613 | 0.966186302 | 0.243380655 | 0.548868866 | 0.838768478 |
| IAM | 0.69545239 | 0.558777756 | 0.351334747 | 0.409395985 | 1.244595698 |
| IAN | 4.733858916 | 3.149119297 | 0.162636174 | 0.171401564 | 1.50323264 |
| ICA | 74.56128894 | 64.93884112 | 0.127220893 | 0.32804226 | 1.148177079 |
| IE | 2.052289671 | 0.504605769 | 0.713196556 | 0.072415677 | 4.067114961 |
| ILA | 1935.7569 | 1084.504961 | 1.073747517 | 0.018547252 | 1.784922126 |
| Indican | 132.9708177 | 163.6299763 | 1.798206542 | 0.039263434 | 0.812631161 |
| IPA | 412.1209144 | 396.65219 | 0.076020528 | 0.951857093 | 1.038998207 |
| IS | 1811.642717 | 766.1269376 | 0.011231967 | 0.188200328 | 2.364676959 |
| KYN | 2980.040274 | 2207.385322 | 1.143852373 | 0.020355161 | 1.350031752 |
| KYNA | 226.2638217 | 59.24751944 | 1.087183033 | 0.093711315 | 3.818958563 |
| Melatonin | 0.534356969 | 0.251453085 | 1.064277668 | 0.004467419 | 2.125076207 |
| Nicotinic acid | 2.355298121 | 3.494814294 | 1.210583448 | 0.056998051 | 0.673940851 |
| NAS | 0.098590597 | 0.152868919 | 1.208305026 | 0.091664399 | 0.64493553 |
| Skatole | 12.33555212 | 14.82829054 | 0.590219239 | 0.110499128 | 0.831893069 |
| Trp | 50369.72727 | 54834.04073 | 1.169899801 | 0.394317641 | 0.918584999 |
| Tryptamine | 0.221192184 | 0.183222803 | 0.313059477 | 0.452938862 | 1.207230654 |
| Xa | 100.9375748 | 101.5191146 | 0.671333463 | 0.949145331 | 0.994271622 |

Table S2-3: Comparison of mean serum concentrations of 28 tryptophans between HE and NC groups.

| **Compound name** | **Mean HE** | **Mean NC** | **VIP** | **P-VALUE** | **FOLD CHANGE** |
| --- | --- | --- | --- | --- | --- |
| 3-HAA | 43.30562567 | 22.07486559 | 0.908216884 | 0.003796387 | 1.961761692 |
| 3-HK | 108.9492639 | 25.95309726 | 1.171176925 | 0.000862602 | 4.197929165 |
| 5-HIAA | 143.0998197 | 63.06782174 | 0.249298662 | 0.137160948 | 2.268983069 |
| 5-HT | 106.3271894 | 576.487183 | 1.801490233 | 7.28532E-09 | 0.184439815 |
| 5-HTOL | 1.597036705 | 0.388687879 | 0.895098301 | 0.009849932 | 4.108789577 |
| 5-HTP | 9.641881481 | 10.75648836 | 0.689961552 | 0.437134477 | 0.896378182 |
| 5-Me-IAA | 0.575917982 | 0.312576983 | 0.81022334 | 0.00358131 | 1.842483654 |
| AA | 25.73229197 | 7.051492211 | 1.392422773 | 0.00013506 | 3.649198098 |
| IA | 1.677324189 | 7.356062995 | 1.089586176 | 0.089892624 | 0.22801928 |
| IAA | 1677.185629 | 2027.012495 | 0.736678584 | 0.196762906 | 0.827417509 |
| IAA-Asp | 0.810406613 | 2.503771371 | 1.020008865 | 0.001718846 | 0.323674367 |
| IAM | 0.69545239 | 0.708962694 | 0.210942156 | 0.957185194 | 0.980943562 |
| IAN | 4.733858916 | 2.113546604 | 0.520850609 | 0.020916547 | 2.239770303 |
| ICA | 74.56128894 | 95.29989697 | 0.89123191 | 0.068772799 | 0.78238583 |
| IE | 2.052289671 | 0.231481645 | 0.725747106 | 0.033363241 | 8.865885115 |
| ILA | 1935.7569 | 951.6583913 | 0.497238273 | 0.005528639 | 2.034087985 |
| Indican | 132.9708177 | 222.0054694 | 1.372465457 | 8.44347E-07 | 0.598952891 |
| IPA | 412.1209144 | 2704.21881 | 1.379993424 | 0.013023197 | 0.152399248 |
| IS | 1811.642717 | 4004.423995 | 1.237915537 | 0.043446405 | 0.452410314 |
| KYN | 2980.040274 | 1898.551844 | 0.646708857 | 0.00076541 | 1.569638608 |
| KYNA | 226.2638217 | 45.68366565 | 0.364845896 | 0.070472937 | 4.95283858 |
| Melatonin | 0.534356969 | 0.043426903 | 1.313486133 | 3.24357E-06 | 12.30474497 |
| Nicotinic acid | 2.355298121 | 1.981427648 | 0.073330539 | 0.374570795 | 1.188687421 |
| NAS | 0.098590597 | 0.309939586 | 1.338295193 | 1.09072E-08 | 0.318096176 |
| Skatole | 12.33555212 | 16.9930479 | 0.813582125 | 0.001966617 | 0.725917575 |
| Trp | 50369.72727 | 66415.04192 | 1.252036843 | 0.000439753 | 0.758408424 |
| Tryptamine | 0.221192184 | 0.469252178 | 1.057557338 | 0.000395918 | 0.471371673 |
| Xa | 100.9375748 | 124.1776562 | 1.107996607 | 0.005418029 | 0.812848124 |

Table S2-4: Comparison of mean serum concentrations of 28 tryptophans between Cir and NC groups.

| **Compound name** | **Mean Cir** | **Mean NC** | **VIP** | **P-VALUE** | **FOLD CHANGE** |
| --- | --- | --- | --- | --- | --- |
| 3-HAA | 33.86680581 | 22.07486559 | 0.465599306 | 0.034445952 | 1.53417948 |
| 3-HK | 49.67311544 | 25.95309726 | 1.085430662 | 0.003514942 | 1.913957126 |
| 5-HIAA | 56.38231512 | 63.06782174 | 0.357368715 | 0.361212076 | 0.893994966 |
| 5-HT | 181.0425459 | 576.487183 | 1.570324985 | 5.08337E-09 | 0.314044356 |
| 5-HTOL | 0.611750862 | 0.388687879 | 0.163770633 | 0.239901468 | 1.573887162 |
| 5-HTP | 8.317155414 | 10.75648836 | 0.724590432 | 0.030460398 | 0.773222183 |
| 5-Me-IAA | 0.326361181 | 0.312576983 | 0.146829607 | 0.659917071 | 1.044098568 |
| AA | 16.32642128 | 7.051492211 | 1.238779958 | 0.012007031 | 2.315314375 |
| IA | 0.954832049 | 7.356062995 | 1.449508614 | 0.053282505 | 0.129802049 |
| IAA | 1348.875703 | 2027.012495 | 1.130915865 | 0.004364017 | 0.665450117 |
| IAA-Asp | 0.966186302 | 2.503771371 | 1.172504299 | 0.004125408 | 0.385892383 |
| IAM | 0.558777756 | 0.708962694 | 0.347647225 | 0.542308901 | 0.788162424 |
| IAN | 3.149119297 | 2.113546604 | 0.806358257 | 0.016486097 | 1.489969179 |
| ICA | 64.93884112 | 95.29989697 | 0.948363982 | 0.001251338 | 0.681415649 |
| IE | 0.504605769 | 0.231481645 | 0.0806911 | 0.156926191 | 2.17989538 |
| ILA | 1084.504961 | 951.6583913 | 0.155725162 | 0.405461029 | 1.139594807 |
| Indican | 163.6299763 | 222.0054694 | 1.29543268 | 0.000646283 | 0.737053807 |
| IPA | 396.65219 | 2704.21881 | 1.733286115 | 0.01210404 | 0.146679029 |
| IS | 766.1269376 | 4004.423995 | 1.62704858 | 5.86228E-05 | 0.191320135 |
| KYN | 2207.385322 | 1898.551844 | 0.629083265 | 0.115236496 | 1.162667919 |
| KYNA | 59.24751944 | 45.68366565 | 0.310278214 | 0.139114999 | 1.296908175 |
| Melatonin | 0.251453085 | 0.043426903 | 1.318353039 | 1.21811E-05 | 5.790260571 |
| Nicotinic acid | 3.494814294 | 1.981427648 | 0.58082871 | 0.003865856 | 1.763785975 |
| NAS | 0.152868919 | 0.309939586 | 1.303925979 | 2.05743E-05 | 0.493221665 |
| Skatole | 14.82829054 | 16.9930479 | 0.606367773 | 0.247416074 | 0.872609236 |
| Trp | 54834.04073 | 66415.04192 | 0.920743852 | 0.007477599 | 0.825626833 |
| Tryptamine | 0.183222803 | 0.469252178 | 1.263076778 | 3.05402E-06 | 0.390457011 |
| Xa | 101.5191146 | 124.1776562 | 1.019417447 | 0.000826864 | 0.817531251 |

Table S3: The faecal concentrations of 11 SCFAs in the three groups.

Table S3-1: Comparison of mean faecal concentrations of 11 SCFAs in the three groups.

| **Compound name** | **Mean NC** | **Mean HE** | **Mean Cir** | **ANOVA P-VALUE** |
| --- | --- | --- | --- | --- |
| Acetic acid | 1151.573708 | 399.8498852 | 390.1456002 | 1.17257E-08 |
| Propionic acid | 1064.869105 | 325.3926249 | 364.077104 | 1.98379E-08 |
| Isobutyric acid | 83.34442288 | 66.25632522 | 23.77084812 | 0.05324766 |
| Butyric acid | 1000.263809 | 477.6469928 | 226.6316784 | 0.001148754 |
| Isovaleric acid | 78.14177883 | 86.76757132 | 26.35834632 | 0.136995693 |
| Valeric acid | 135.322919 | 88.88724253 | 21.44245346 | 0.137615495 |
| Hexanoic acid | 24.47024297 | 47.2943584 | 2.581844932 | 0.393686569 |
| Heptanoicacid | 1.742023148 | 5.533858611 | 0.085366634 | 0.400985093 |
| Octanoic acid | 0.64499171 | 4.060148891 | 0.452403672 | 0.223112698 |
| Nonanoic acid | 6.244537828 | 7.673836592 | 6.591226077 | 0.047466555 |
| Decanoic acid | 0.348373962 | 2.863333961 | 0.737479104 | 0.103085756 |

Table S3-2: Comparison of mean faecal concentrations of 11 SCFAs between HE and Cir groups.

| **Compound name** | **Mean HE** | **Mean Cir** | **VIP** | **P-VALUE** | **FOLD CHANGE** |
| --- | --- | --- | --- | --- | --- |
| Acetic acid | 399.8498852 | 390.1456002 | 0.377145105 | 0.936370894 | 1.024873496 |
| Propionic acid | 325.3926249 | 364.077104 | 0.627341622 | 0.7291779 | 0.893746466 |
| Isobutyric acid | 66.25632522 | 23.77084812 | 0.085220654 | 0.095791208 | 2.78729328 |
| Butyric acid | 477.6469928 | 226.6316784 | 0.213733296 | 0.214241123 | 2.107591472 |
| Isovaleric acid | 86.76757132 | 26.35834632 | 0.110642043 | 0.092909902 | 3.291844271 |
| Valeric acid | 88.88724253 | 21.44245346 | 0.423015299 | 0.244018352 | 4.14538582 |
| Hexanoic acid | 47.2943584 | 2.581844932 | 1.477898842 | 0.232128917 | 18.31804762 |
| Heptanoic acid | 5.533858611 | 0.085366634 | 1.463888369 | 0.252997184 | 64.82460811 |
| Octanoic acid | 4.060148891 | 0.452403672 | 1.635113318 | 0.175506302 | 8.974615254 |
| Nonanoic acid | 7.673836592 | 6.591226077 | 1.318287258 | 0.067268136 | 1.164250248 |
| Decanoic acid | 2.863333961 | 0.737479104 | 1.217146241 | 0.131331394 | 3.882596736 |

Table S3-3: Comparison of mean faecal concentrations of 11 SCFAs between HE and NC groups.

| **Compound name** | **Mean HE** | **Mean NC** | **VIP** | **P-VALUE** | **FOLD CHANGE** |
| --- | --- | --- | --- | --- | --- |
| Acetic acid | 399.8498852 | 1151.573708 | 1.352942049 | 8.50426E-08 | 0.347220401 |
| Propionic acid | 325.3926249 | 1064.869105 | 1.431061175 | 1.08257E-07 | 0.305570538 |
| Isobutyric acid | 66.25632522 | 83.34442288 | 1.206488181 | 0.547023218 | 0.794970112 |
| Butyric acid | 477.6469928 | 1000.263809 | 1.30159458 | 0.018328769 | 0.477521018 |
| Isovaleric acid | 86.76757132 | 78.14177883 | 1.187594465 | 0.820619274 | 1.110386436 |
| Valeric acid | 88.88724253 | 135.322919 | 1.05457012 | 0.465828625 | 0.656852832 |
| Hexanoic acid | 47.2943584 | 24.47024297 | 0.166768228 | 0.558046175 | 1.932729416 |
| Heptanoic acid | 5.533858611 | 1.742023148 | 0.255696239 | 0.430352506 | 3.17668489 |
| Octanoic acid | 4.060148891 | 0.64499171 | 0.556493506 | 0.200344909 | 6.294885391 |
| Nonanoic acid | 7.673836592 | 6.244537828 | 0.755316669 | 0.018265093 | 1.228887838 |
| Decanoic acid | 2.863333961 | 0.348373962 | 0.689906001 | 0.07404958 | 8.219138846 |

Table S3-4: Comparison of mean faecal concentrations of 11 SCFAs between Cir and NC groups.

| **Compound name** | **Mean Cir** | **Mean NC** | **VIP** | **P-VALUE** | **FOLD CHANGE** |
| --- | --- | --- | --- | --- | --- |
| Acetic acid | 390.1456002 | 1151.573708 | 1.1821151 | 2.9211E-10 | 0.338793425 |
| Propionic acid | 364.077104 | 1064.869105 | 1.164666475 | 2.36754E-07 | 0.341898457 |
| Isobutyric acid | 23.77084812 | 83.34442288 | 1.191555716 | 0.001230816 | 0.285212223 |
| Butyric acid | 226.6316784 | 1000.263809 | 1.265794662 | 1.63056E-09 | 0.226571907 |
| Isovaleric acid | 26.35834632 | 78.14177883 | 1.128545147 | 0.009420065 | 0.337314388 |
| Valeric acid | 21.44245346 | 135.322919 | 1.28086611 | 0.000897723 | 0.158453968 |
| Hexanoic acid | 2.581844932 | 24.47024297 | 1.096511116 | 0.087259758 | 0.105509575 |
| Heptanoicacid | 0.085366634 | 1.742023148 | 0.851195074 | 0.061177926 | 0.049004305 |
| Octanoic acid | 0.452403672 | 0.64499171 | 0.15244039 | 0.45518844 | 0.701410056 |
| Nonanoic acid | 6.591226077 | 6.244537828 | 0.438676649 | 0.215469943 | 1.05551864 |
| Decanoic acid | 0.737479104 | 0.348373962 | 0.409069242 | 0.062200406 | 2.116917982 |

Table S4 The faecal concentrations of 29 tryptophan metabolites in the three groups.

Table S4-1 Comparison of mean faecal concentrations of 29 tryptophans in the three groups.

| **Compound name** | **Mean HE** | **Mean Cir** | **Mean NC** | **ANOVA P-VALUE** |
| --- | --- | --- | --- | --- |
| 3-HAA | 139.5303548 | 420.496401 | 339.5425286 | 0.454765752 |
| 3-HK | 161.9787646 | 422.466353 | 190.774683 | 0.555796803 |
| 5-HIAA | 664.3683927 | 1843.526891 | 1788.81457 | 0.419509894 |
| 5-HT | 1268.977756 | 957.4114311 | 4198.176908 | 0.187209829 |
| 5-HTOL | 210.691265 | 227.01157 | 91.20465175 | 0.531175278 |
| 5-HTP | 48.64067342 | 75.32930334 | 16.22176666 | 0.040368959 |
| 5-Me-IAA | 0.992538305 | 1.208043384 | 0.17897307 | 0.001120536 |
| AA | 156.7145369 | 158.1271942 | 247.892462 | 0.296099344 |
| IA | 14.00317747 | 21.26299784 | 102.7122218 | 0.011684066 |
| IAA | 3487.576269 | 2989.90013 | 6011.671606 | 0.010637368 |
| IAA-Ala | 1.256510958 | 4.346424692 | 0.836153056 | 0.060133034 |
| IAA-Asp | 141.4641597 | 130.8792895 | 56.44222482 | 0.622013592 |
| IAM | 25.58633695 | 12.43579728 | 10.17570955 | 0.568976898 |
| IAN | 2415.724443 | 492.1623242 | 604.9960023 | 0.304638486 |
| ICA | 357.1583305 | 375.6019037 | 497.2923582 | 0.309596467 |
| IE | 231.169801 | 167.7000886 | 276.4017288 | 0.361974215 |
| IGA | 74.10570439 | 100.9046692 | 52.96675839 | 0.297040329 |
| ILA | 12473.7168 | 13166.45334 | 1152.286472 | 0.24654815 |
| Indole | 731.8103177 | 331.621335 | 358.2980868 | 0.525927685 |
| IPA | 2308.987728 | 2871.148806 | 9807.832508 | 8.18135E-05 |
| IS | 587.5694527 | 7682.721555 | 1304.68803 | 0.446839887 |
| KYN | 573.2260237 | 1478.782124 | 406.8395359 | 0.215797319 |
| KYNA | 5457.836735 | 3584.826688 | 6195.160821 | 0.397929427 |
| Nicotinic acid | 22677.76653 | 23348.226 | 76981.35372 | 5.10357E-05 |
| NAS | 8.717893797 | 7.639352121 | 9.346031352 | 0.965093529 |
| Skatole | 452.8911672 | 615.4072993 | 731.1279188 | 0.582570182 |
| Trp | 421593.423 | 400898.8972 | 42381.44741 | 3.43835E-06 |
| Tryptamine | 5048.651113 | 5379.647521 | 4283.377982 | 0.907107309 |
| Xa | 1061.266687 | 1078.621239 | 244.6214305 | 0.006363792 |

Table S4-2 Comparison of mean faecal concentrations of 29 tryptophans between HE and Cir groups.

| **Compound name** | **Mean HE** | **Mean Cir** | **VIP** | **P-VALUE** | **FOLD CHANGE** |
| --- | --- | --- | --- | --- | --- |
| 3-HAA | 139.5303548 | 420.496401 | 0.814176249 | 0.281194333 | 0.331822947 |
| 3-HK | 161.9787646 | 422.466353 | 1.670578939 | 0.384068149 | 0.383412226 |
| 5-HIAA | 664.3683927 | 1843.526891 | 0.863165989 | 0.283228398 | 0.360379008 |
| 5-HT | 1268.977756 | 957.4114311 | 0.043868813 | 0.502250777 | 1.325425742 |
| 5-HTOL | 210.691265 | 227.01157 | 1.384062561 | 0.899487098 | 0.928108048 |
| 5-HTP | 48.64067342 | 75.32930334 | 0.782234206 | 0.262742664 | 0.645707198 |
| 5-Me-IAA | 0.992538305 | 1.208043384 | 0.641470705 | 0.630421059 | 0.821608162 |
| AA | 156.7145369 | 158.1271942 | 1.629055589 | 0.979882955 | 0.991066323 |
| IA | 14.00317747 | 21.26299784 | 1.030572392 | 0.36213965 | 0.658570234 |
| IAA | 3487.576269 | 2989.90013 | 0.137457264 | 0.581462333 | 1.166452429 |
| IAA-Ala | 1.256510958 | 4.346424692 | 1.993498322 | 0.049083133 | 0.2890907 |
| IAA-Asp | 141.4641597 | 130.8792895 | 0.448527316 | 0.9073539 | 1.080875058 |
| IAM | 25.58633695 | 12.43579728 | 1.098131638 | 0.435430017 | 2.057474594 |
| IAN | 2415.724443 | 492.1623242 | 0.072329742 | 0.218284751 | 4.90838962 |
| ICA | 357.1583305 | 375.6019037 | 0.336975325 | 0.844896689 | 0.950895954 |
| IE | 231.169801 | 167.7000886 | 1.25115876 | 0.350517198 | 1.378471549 |
| IGA | 74.10570439 | 100.9046692 | 1.607331594 | 0.380172184 | 0.734413035 |
| ILA | 12473.7168 | 13166.45334 | 0.34946663 | 0.931091918 | 0.947386246 |
| Indole | 731.8103177 | 331.621335 | 2.105341773 | 0.361560785 | 2.206764886 |
| IPA | 2308.987728 | 2871.148806 | 0.403433495 | 0.695326233 | 0.804203434 |
| IS | 587.5694527 | 7682.721555 | 0.127397728 | 0.311091257 | 0.076479337 |
| KYN | 573.2260237 | 1478.782124 | 1.40053914 | 0.212705645 | 0.387633861 |
| KYNA | 5457.836735 | 3584.826688 | 0.426055449 | 0.360839577 | 1.522482733 |
| Nicotinic acid | 22677.76653 | 23348.226 | 0.030877326 | 0.923217618 | 0.971284351 |
| NAS | 8.717893797 | 7.639352121 | 0.413924851 | 0.869895595 | 1.141182349 |
| Skatole | 452.8911672 | 615.4072993 | 0.640177889 | 0.479655733 | 0.73592102 |
| Trp | 421593.423 | 400898.8972 | 0.949048564 | 0.86839569 | 1.051620311 |
| Tryptamine | 5048.651113 | 5379.647521 | 0.233150163 | 0.889290568 | 0.938472473 |
| Xa | 1061.266687 | 1078.621239 | 0.250839449 | 0.951898374 | 0.98391043 |

Table S4-3 Comparison of mean faecal concentrations of 29 tryptophans between HE and NC groups.

| **Compound name** | **Mean HE** | **Mean NC** | **VIP** | **P-VALUE** | **FOLD CHANGE** |
| --- | --- | --- | --- | --- | --- |
| 3-HAA | 139.5303548 | 339.5425286 | 0.878096021 | 0.046517621 | 0.410936313 |
| 3-HK | 161.9787646 | 190.774683 | 0.920044746 | 0.645535935 | 0.849057968 |
| 5-HIAA | 664.3683927 | 1788.81457 | 0.89181149 | 0.035183421 | 0.371401488 |
| 5-HT | 1268.977756 | 4198.176908 | 0.358091971 | 0.319226353 | 0.302268767 |
| 5-HTOL | 210.691265 | 91.20465175 | 0.232692308 | 0.230570743 | 2.31009341 |
| 5-HTP | 48.64067342 | 16.22176666 | 1.472432435 | 0.000476122 | 2.998481881 |
| 5-Me-IAA | 0.992538305 | 0.17897307 | 1.053858004 | 0.02695142 | 5.545741088 |
| AA | 156.7145369 | 247.892462 | 1.251803523 | 0.185383796 | 0.632187585 |
| IA | 14.00317747 | 102.7122218 | 1.3566894 | 0.005437533 | 0.136334092 |
| IAA | 3487.576269 | 6011.671606 | 1.099193941 | 0.020346378 | 0.580134195 |
| IAA-Ala | 1.256510958 | 0.836153056 | 0.489234395 | 0.255511151 | 1.502728416 |
| IAA-Asp | 141.4641597 | 56.44222482 | 0.835442 | 0.364034222 | 2.506353357 |
| IAM | 25.58633695 | 10.17570955 | 0.664160033 | 0.374647545 | 2.514452365 |
| IAN | 2415.724443 | 604.9960023 | 0.004849072 | 0.260449276 | 3.992959349 |
| ICA | 357.1583305 | 497.2923582 | 0.986509478 | 0.089133538 | 0.71820595 |
| IE | 231.169801 | 276.4017288 | 0.206118048 | 0.611095735 | 0.836354396 |
| IGA | 74.10570439 | 52.96675839 | 0.445303759 | 0.381041819 | 1.399098352 |
| ILA | 12473.7168 | 1152.286472 | 1.403308331 | 0.006385857 | 10.82518723 |
| Indole | 731.8103177 | 358.2980868 | 0.188964108 | 0.380894048 | 2.042462253 |
| IPA | 2308.987728 | 9807.832508 | 1.845899191 | 0.000382227 | 0.235422834 |
| IS | 587.5694527 | 1304.68803 | 0.372629901 | 0.346723847 | 0.450352451 |
| KYN | 573.2260237 | 406.8395359 | 0.853866298 | 0.322067649 | 1.408973251 |
| KYNA | 5457.836735 | 6195.160821 | 0.804819706 | 0.702987789 | 0.880983867 |
| Nicotinic acid | 22677.76653 | 76981.35372 | 1.410516921 | 0.005201598 | 0.294587786 |
| NAS | 8.717893797 | 9.346031352 | 0.873764678 | 0.927525425 | 0.932790986 |
| Skatole | 452.8911672 | 731.1279188 | 0.738859354 | 0.320440289 | 0.619441763 |
| Trp | 421593.423 | 42381.44741 | 1.817026295 | 4.13738E-05 | 9.947593789 |
| Tryptamine | 5048.651113 | 4283.377982 | 0.056010611 | 0.763641751 | 1.178661126 |
| Xa | 1061.266687 | 244.6214305 | 1.544719056 | 1.49607E-05 | 4.338404385 |

Table S4-4 Comparison of mean faecal concentrations of 29 tryptophans between Cir and NC groups.

| **Compound name** | **Mean Cir** | **Mean NC** | **VIP** | **P-VALUE** | **FOLD CHANGE** |
| --- | --- | --- | --- | --- | --- |
| 3-HAA | 420.496401 | 339.5425286 | 0.607257294 | 0.7627691 | 1.23842042 |
| 3-HK | 422.466353 | 190.774683 | 0.539849085 | 0.435784657 | 2.214478076 |
| 5-HIAA | 1843.526891 | 1788.81457 | 0.59290889 | 0.963127766 | 1.030585798 |
| 5-HT | 957.4114311 | 4198.176908 | 0.557514283 | 0.26845407 | 0.228054094 |
| 5-HTOL | 227.01157 | 91.20465175 | 0.156703613 | 0.183414887 | 2.489034996 |
| 5-HTP | 75.32930334 | 16.22176666 | 1.576627546 | 0.013335334 | 4.643717598 |
| 5-Me-IAA | 1.208043384 | 0.17897307 | 1.327742774 | 0.001563918 | 6.749861238 |
| AA | 158.1271942 | 247.892462 | 1.200452648 | 0.160834343 | 0.637886255 |
| IA | 21.26299784 | 102.7122218 | 1.391432726 | 0.010436915 | 0.207015265 |
| IAA | 2989.90013 | 6011.671606 | 1.054764906 | 0.003637106 | 0.497349211 |
| IAA-Ala | 4.346424692 | 0.836153056 | 0.982294758 | 0.027288846 | 5.198120917 |
| IAA-Asp | 130.8792895 | 56.44222482 | 0.620209306 | 0.210118233 | 2.318818755 |
| IAM | 12.43579728 | 10.17570955 | 0.280598878 | 0.758834958 | 1.222106155 |
| IAN | 492.1623242 | 604.9960023 | 0.063460171 | 0.823577552 | 0.81349682 |
| ICA | 375.6019037 | 497.2923582 | 0.867978544 | 0.140230104 | 0.755293938 |
| IE | 167.7000886 | 276.4017288 | 0.503251617 | 0.155187538 | 0.606725903 |
| IGA | 100.9046692 | 52.96675839 | 0.652610416 | 0.118618819 | 1.905056535 |
| ILA | 13166.45334 | 1152.286472 | 1.564075019 | 0.097118993 | 11.42637153 |
| Indole | 331.621335 | 358.2980868 | 0.678427784 | 0.898161741 | 0.925545927 |
| IPA | 2871.148806 | 9807.832508 | 1.932821756 | 0.001077395 | 0.292740399 |
| IS | 7682.721555 | 1304.68803 | 0.474259489 | 0.363638728 | 5.888550656 |
| KYN | 1478.782124 | 406.8395359 | 1.126587905 | 0.140802044 | 3.634804373 |
| KYNA | 3584.826688 | 6195.160821 | 1.066368364 | 0.103540244 | 0.578649496 |
| Nicotinic acid | 23348.226 | 76981.35372 | 1.623022163 | 0.005667418 | 0.303297161 |
| NAS | 7.639352121 | 9.346031352 | 0.640991473 | 0.664369202 | 0.817389952 |
| Skatole | 615.4072993 | 731.1279188 | 0.621123493 | 0.717908821 | 0.841723156 |
| Trp | 400898.8972 | 42381.44741 | 0.936609192 | 0.000929929 | 9.459301692 |
| Tryptamine | 5379.647521 | 4283.377982 | 0.146080845 | 0.63214236 | 1.255935746 |
| Xa | 1078.621239 | 244.6214305 | 1.564145513 | 0.002180675 | 4.409348914 |

Table S5 Tryptophan metabolites list and abbreviation.

| **NO.** | **Full name** | **Abbreviation** |
| --- | --- | --- |
| 1 | Indole | / |
| 2 | Nicotinic acid | NA |
| 3 | Skatole | / |
| 4 | Anthranilic acid | AA |
| 5 | Indole-3-carboxaldehyde | ICA |
| 6 | Indole-3-acetonitrile | IAN |
| 7 | 3-Hydroxyanthranilic acid | 3-HAA |
| 8 | Tryptamine | / |
| 9 | Indole ethanol/tryptophol | IE |
| 10 | Indole-3-acetamide | IAM |
| 11 | Indole-3-acetic acid | IAA |
| 12 | Serotonin | 5-HT |
| 13 | 5-Hydroxytryptophol | 5-HTOL |
| 14 | Indole acrylic acid | IA |
| 15 | 3-Indoleglyoxylic acid | IGA |
| 16 | Kynurenic acid | KYNA |
| 17 | 3-Indolepropionic acid | IPA |
| 18 | 5-Hydroxyindoleacetic acid | 5-HIAA |
| 19 | L-Tryptophan | Trp |
| 20 | Xanthurenic acid | Xa |
| 21 | Indolelactic acid | ILA |
| 22 | 5-Methoxy-3-indoleacetic | 5-Me-IAA |
| 23 | Kynurenine | KYN |
| 24 | Indoxylsulfate | IS |
| 25 | N-Acetyl-5-hydroxytryptamine | NAS |
| 26 | L-5-Hydroxytryptophan | 5-HTP |
| 27 | 3-Hydroxykynurenine | 3-HK |
| 28 | Melatonin | / |
| 29 | Indican | / |
| 30 | Indole-3-acetyl-alanine | IAA-Ala |
| 31 | Indole-3-acetyl-aspartate | IAA-Asp |
