# Supplementary material for: Integrative analysis of the gut microbiota and faecal and serum short-chain fatty acids and tryptophan metabolites in patients with cirrhosis and hepatic encephalopathy
Source: J Transl Med. 2023 Jun 17;21:395. doi: 10.1186/s12967-023-04262-9 (PMC10276405; doi:10.1186/s12967-023-04262-9)
Supplement: Supplementary file 2 — Additional file 2: Figure S1 The relative abundances of dominant taxa at class level (A), order level (B), family level (C), and species (D). Figure S2 Analysis result of the function annotationsin Cir group and NC group based on the MetaCyc database. Figure S3 Analysis result of the function annotationsin HE group and NC group based on the MetaCyc database. [file 12967_2023_4262_MOESM2_ESM.docx]

***Additional file 2 (Figure)***


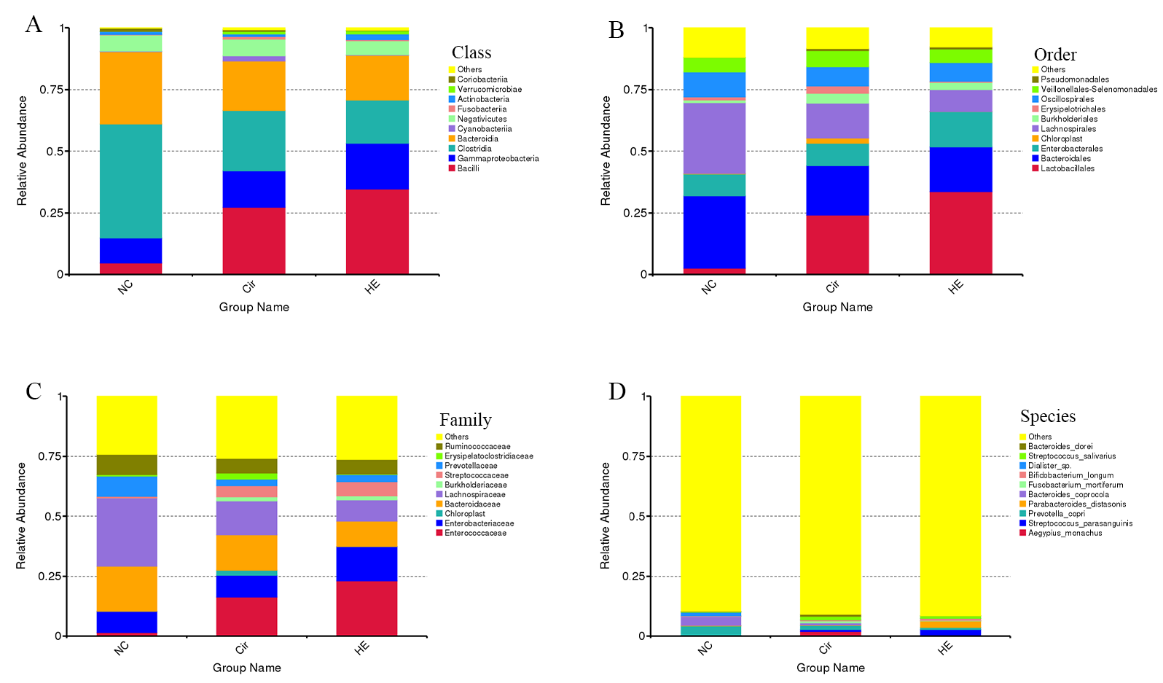


Figure S1 The relative abundances of dominant taxa at class level (A), order level (B), family level (C), and species (D).


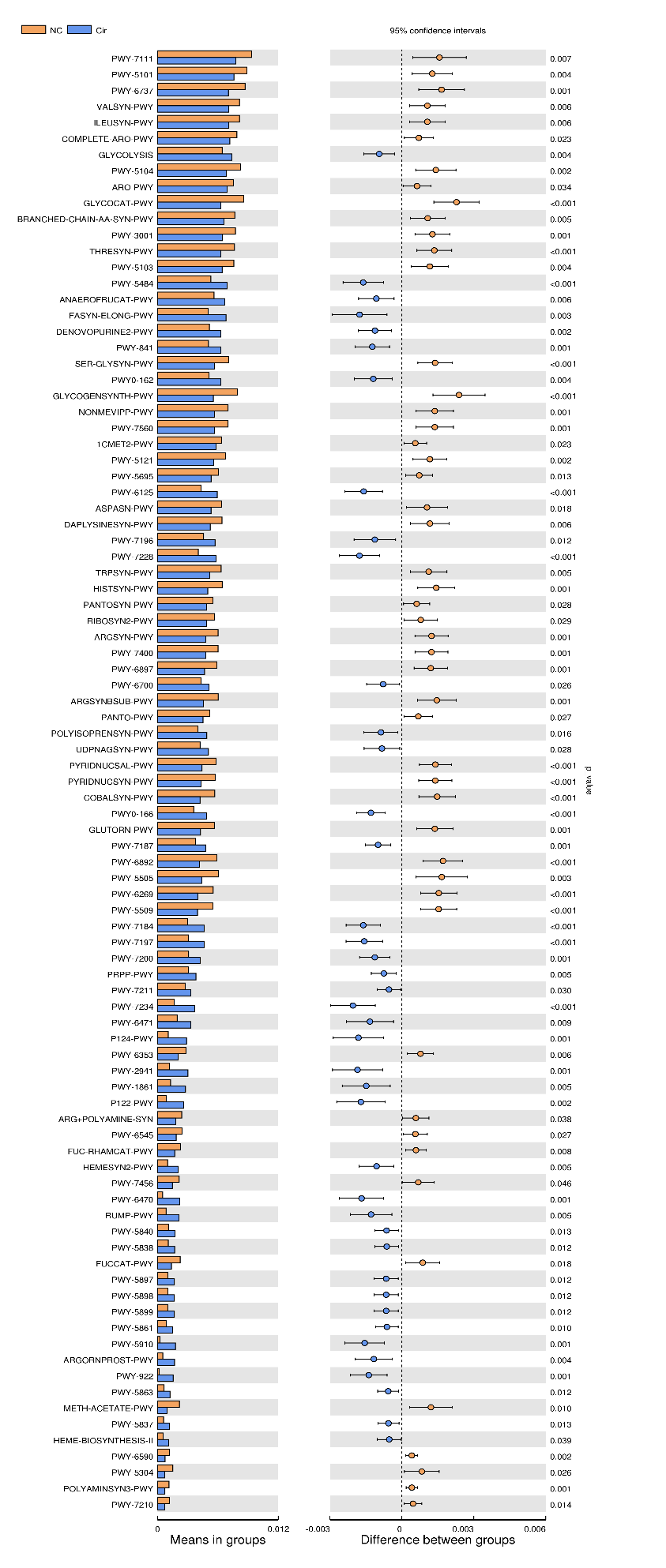


Figure S2 Analysis result of the function annotations in Cir group and NC group based on the MetaCyc database.


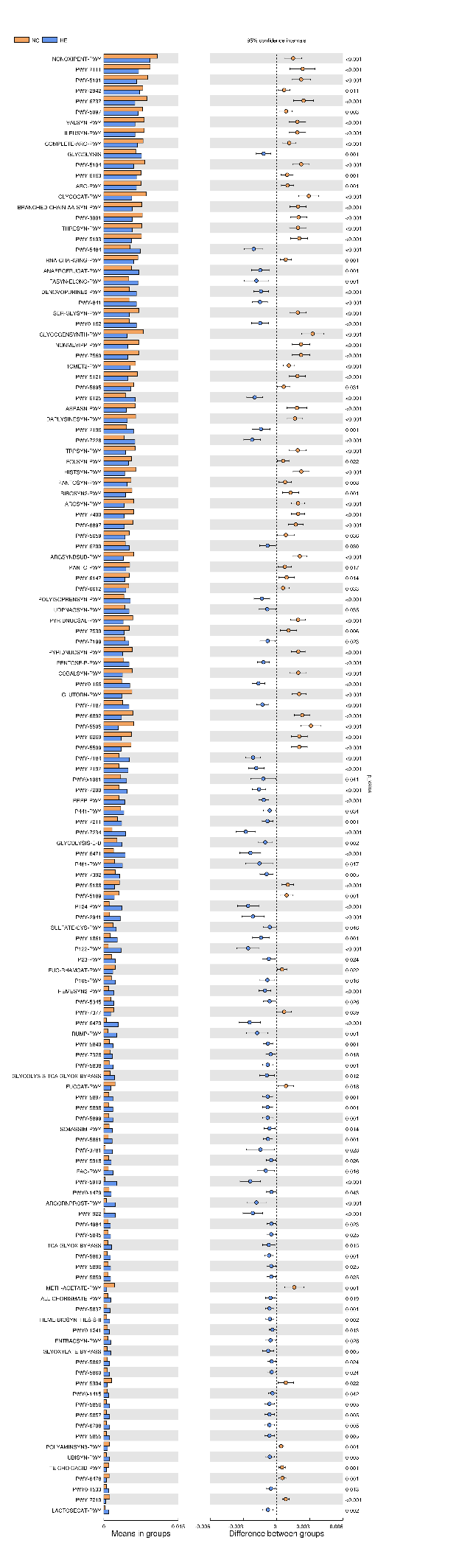


Figure S3 Analysis result of the function annotations in HE group and NC group based on the MetaCyc database.
