# Supplementary material for: Integrative analysis of the gut microbiota and faecal and serum short-chain fatty acids and tryptophan metabolites in patients with cirrhosis and hepatic encephalopathy
Source: J Transl Med. 2023 Jun 17;21:395. doi: 10.1186/s12967-023-04262-9 (PMC10276405; doi:10.1186/s12967-023-04262-9)
Supplement: Supplementary file 3 — Additional file 3. The methods for quantifying the concentrations of SCFAs and tryptophan metabolites. [file 12967_2023_4262_MOESM3_ESM.docx]

***Additional file 3 (Method)***

***(The methods for quantifying the concentrations of SCFAs and tryptophan metabolites)***

**Part 1 GC-MS for serum SCFAs**

1. **Reagents and equipment**

Table1 The list of reagents.

| Name | CAS | Brand |
| --- | --- | --- |
| Methyl tert-butyl ether | 1634-04-4 | CNW |
| Sulfuric acid | 7664-93-9 | China National Pharmaceutical Group Corporation |
| 2-Methylvaleric acid | 97-61-0 | Dr. Ehrenstorfer |
| Acetic acid | 64-19-7 | Dr. Ehrenstorfer |
| Propionic acid | 79-09-4 | Dr. Ehrenstorfer |
| Isobutyric acid | 79-31-2 | Dr. Ehrenstorfer |
| Butyric acid | 107-92-6 | Dr. Ehrenstorfer |
| Isovaleric acid | 503-74-2 | Dr. Ehrenstorfer |
| Valeric acid | 109-52-4 | Dr. Ehrenstorfer |
| Hexanoic acid | 142-62-1 | Dr. Ehrenstorfer |
| Heptanoicacid | 111-14-8 | Dr. Ehrenstorfer |
| Octanoic acid | 124-07-2 | Dr. Ehrenstorfer |
| Nonanoic acid | 112-05-0 | Dr. Ehrenstorfer |
| Decanoic acid | 334-48-5 | Dr. Ehrenstorfer |

Table 2 Laboratory equipment.

| Equipment | Model | Brand |
| --- | --- | --- |
| GC | GC-2030 | SHIMADZU |
| MS | QP2020 NX | SHIMADZU |
| Chromatographic column | HP-FFAP（30m×250μm×0.25μm） | Agilent |
| Centrifuge | Heraeus Fresco17 | Thermo Fisher Scientific |

1. **Method**

**2.1 Metabolites Extraction：**

Take 0.1 mL sample into the 1.5 mL EP tubes, add 0.05 mL50 % H_2_SO_4_ and 0.2 mL of 2-Methylvaleric acid (25 mg/L stock in methyl tert-butyl ether) as internal standard, vortex mixing for 30 s, oscillations in 10 min, then ultrasound treated for 10 min (incubated in ice water). Centrifuge for 15 min at 10000 rpm, 4 °C. Keep at −20 °C for 30 min. Transfer the supernatant in to a fresh 2 mL glass vial, for GC-MS analysis.

**2.2 GC-MS Analysis：**

SHIMADZU GC2030-QP2020 NX gas chromatography-mass spectrometer. The system utilized a HP-FFAP capillary column. A 1 μL aliquot of the analyte was injected in split mode (5:1). Helium was used as the carrier gas, the front inlet purge flow was 3 mL min−1, and the gas flow rate through the column was 1 mL min−1. The initial temperature was kept at 80 °C for 1 min, then raised to 200 °C at a rate of 10 °C min−1 for 5 min, then kept for 1 min at 240 °C at a rate of 40 °C min−1. The injection, transfer line, quad and ion source temperatures were 240 °C, 240 °C, 150 °C and 200 °C. The energy was -70 eV in electron impact mode. The mass spectrometry data were acquired in Scan/SIM mode with the m/z range of 33-150 after a solvent delay of 3.5 min.

**2.3 Standard curve**

| Compound name | Retention time（min） | Linear equation | *R^2^* | Linear range（mg/L） |
| --- | --- | --- | --- | --- |
| Acetic acid | 5.609 | y=0.016x + 0.003 | 0.9998 | 0.5~50 |
| Propionic acid | 6.583 | y=0.0115x + 1E-04 | 0.9997 | 0.02~1 |
| Isobutyric acid | 6.895 | y=0.0372x + 0.0014 | 0.9994 | 0.02~1 |
| Butyric acid | 7.593 | y=0.0397x + 8E-05 | 0.9998 | 0.02~1 |
| Isovaleric acid | 8.052 | y=0.0441x + 0.0001 | 0.9999 | 0.02~1 |
| Valeric acid | 8.819 | y=0.0464x + 0.0002 | 0.9999 | 0.02~1 |
| Hexanoic acid | 9.979 | y=0.0408x + 0.0004 | 0.9999 | 0.02~1 |
| Heptanoicacid | 11.085 | y=0.0385x + 0.0001 | 1.0000 | 0.02~1 |
| Octanoic acid | 12.147 | y=0.0334x + 0.0002 | 1.0000 | 0.02~1 |
| Nonanoic acid | 13.161 | y=0.0289x + 0.0002 | 0.9998 | 0.02~1 |
| Decanoic acid | 14.296 | y=0.027x + 3E-05 | 0.9999 | 0.02~1 |

**2.4 Calculation formula：**

C（con）=$\frac{Cs*V1}{\begin{aligned} V0 \\ \end{aligned}}$

C(con): the amount of the target compound in the sample (μg /mL)；

Cs: concentration of target compound in extract (mg/L)；

V1: the extraction solution volume (mL)；

V0: sample volume (mL).

**Part 2 GC-MS for fecal SCFAs**

1. **Reagents and equipment**

The reagents and equipment utilized for detecting fecal SCFAs are identical to those used for detecting serum SCFAs.

1. **Method**
   1. **Metabolites Extraction：**

Take sample into the 2 mL EP tubes, extracted with 1 mL H2O, vortex mixing for 10 s. Homogenized in ball mill for 4 min at 40 Hz, then ultrasound treated for 5 min (incubated in ice water)，repeat 3 times. Centrifuge for 20 min at 5000 rpm, 4 °C. Transfer the supernatant 0.8 mL into a fresh 2 mL EP tubes; Add 0.1 mL 50 % H2SO4 and 0.8 mL of extracting solution (25 mg/L stock in methyl tert-butyl ether) as internal standard, vortex mixing for 10 s, oscillations in 10 min, then ultrasound treated for 10 min (incubated in ice water). Centrifuge for 15 min at 10000 rpm, 4 °C. Keep at −20 °C for 30 min. Transfer the supernatant into a fresh 2 mL glass vial, for GC-MS analysis.

**2.2 GC-MS Analysis：**

SHIMADZU GC2030-QP2020 NX gas chromatography-mass spectrometer. The system utilized a HP-FFAP capillary column. A 1 μL aliquot of the analyte was injected in split mode (5:1). Helium was used as the carrier gas, the front inlet purge flow was 3 mL min^−1^, and the gas flow rate through the column was 1 mL min^−1^. The initial temperature was kept at 80 °C for 1 min, then raised to 200 °C at a rate of 10 °C min^−1^ for 5 min, then kept for 1 min at 240 °C at a rate of 40 °C min^−1^. The injection, transfer line, quad and ion source temperatures were 240 °C, 240 °C, 150 °C and 200 °C. The energy was -70 eV in electron impact mode. The mass spectrometry data were acquired in Scan/SIM mode with the m/z range of 33-150 after a solvent delay of 3.5 min.

- 1. **Standard curve**

| Compound name | Retention time（min） | Linear equation | *R^2^* | Linear range（mg/L） |
| --- | --- | --- | --- | --- |
| Acetic acid | 5.380 | y=0.0151x - 0.001 | 0.9995 | 0.5~100 |
| Propionic acid | 6.350 | y=0.0115x – 0.0009 | 0.9995 | 0.02~100 |
| Isobutyric acid | 6.670 | y=0.0291x + 0.0019 | 0.9999 | 0.02~50 |
| Butyric acid | 7.365 | y=0.0395x + 0.0006 | 0.9998 | 0.02~100 |
| Isovaleric acid | 7.835 | y=0.0439x - 0.0002 | 1.0000 | 0.02~100 |
| Valeric acid | 8.595 | y=0.0476x - 0.0051 | 0.9997 | 0.02~100 |
| Hexanoic acid | 9.760 | y=0.0419x - 0.0030 | 0.9997 | 0.02~100 |
| Heptanoicacid | 10.875 | y=0.0444x + 0.0003 | 0.9998 | 0.02~1 |
| Octanoic acid | 11.945 | y=0.0386x + 0.0003 | 0.9997 | 0.02~1 |
| Nonanoic acid | 12.960 | y=0.033x + 0.0002 | 0.9994 | 0.02~1 |
| Decanoic acid | 14.055 | y=0.0306x + 8E-05 | 0.9999 | 0.02~1 |

**2.4 Calculation formula：**

C(con）=$\frac{\mathrm{Cs}*V1*V3}{M*V2}$*1000

C(con): the amount of the target compound in the sample (μg /g)；

Cs: concentration of target compound in extract (mg/L)；

V1: the extraction solution volume (mL)；

V2: volume removed from ddH_2_O supernatant (mL);

V3: the volume of ddH_2_O added (mL)；

M: sample weight (mg).

**Part 3 UHPLC-MS-MS for serum tryptophan metabolites**

1. **Reagents and equipment**

Table1 The list of reagents.

| Name | CAS | Brand |
| --- | --- | --- |
| Methanol | 67-56-1 | CNW Technologies |
| Acetonitrile | 75-05-8 | CNW Technologies |
| Formic acid | 64-18-6 | CNW Technologies |

Table 2 The list of tryptophan metabolites

| **Compound name** | **Abbreviation.** | **CAS** | **Brand** |
| --- | --- | --- | --- |
| 3-Hydroxyanthranilic acid | 3-HAA | 548-93-6 | Dr. Ehrenstorfer |
| 3-Hydroxykynurenine | 3-HK | 2147-61-7 | Dr. Ehrenstorfer |
| 5-Hydroxyindoleacetic acid | 5-HIAA | 54-16-0 | Dr. Ehrenstorfer |
| Serotonin | 5-HT | 50-67-9 | Dr. Ehrenstorfer |
| 5-Hydroxytryptophol | 5-HTOL | 154-02-9 | Dr. Ehrenstorfer |
| L-5-Hydroxytryptophan | 5-HTP | 4350-09-8 | Dr. Ehrenstorfer |
| 5-Methoxy-3-indoleacetic acid | 5-Me-IAA | 3471-31-6 | Dr. Ehrenstorfer |
| Anthranilic acid | AA | 118-92-3 | Dr. Ehrenstorfer |
| Indole acrylic acid | IA | 29953-71-7 | Dr. Ehrenstorfer |
| Indole-3-acetic acid | IAA | 87-51-4 | Dr. Ehrenstorfer |
| Indole-3-acetamide | IAM | 879-37-8 | Dr. Ehrenstorfer |
| Indole-3-carboxaldehyde | ICA | 487-89-8 | Dr. Ehrenstorfer |
| Indole ethanol/tryptophol | IE | 526-55-6 | Dr. Ehrenstorfer |
| 3-Indoleglyoxylic acid | IGA | 1477-49-2 | Dr. Ehrenstorfer |
| Indolelactic acid | ILA | 1821-52-9 | Dr. Ehrenstorfer |
| Indican | Indican | 487-60-5 | Dr. Ehrenstorfer |
| Indole | Indole | 120-72-9 | Dr. Ehrenstorfer |
| 3-Indolepropionic acid | IPA | 830-96-6 | Dr. Ehrenstorfer |
| Indoxylsulfate | IS | 2642-37-7 | Dr. Ehrenstorfer |
| Kynurenine | KYN | 343-65-7 | Dr. Ehrenstorfer |
| Kynurenic acid | KYNA | 492-27-3 | Dr. Ehrenstorfer |
| Melatonin | Melatonin | 73-31-4 | Dr. Ehrenstorfer |
| Nicotinic acid | NA | 59-67-6 | Dr. Ehrenstorfer |
| N-Acetyl-5-hydroxytryptamine | NAS | 1210-83-9 | Dr. Ehrenstorfer |
| Skatole | skatole | 83-34-1 | Dr. Ehrenstorfer |
| L-Tryptophan | Trp | 73-22-3 | Dr. Ehrenstorfer |
| Tryptamine | Tryptamine | 61-54-1 | Dr. Ehrenstorfer |
| Xanthurenic acid | Xa | 59-00-7 | Dr. Ehrenstorfer |
| Indole-3-acetonitrile | IAN | 771-51-7 | Dr. Ehrenstorfer |
| Indole-3-acetyl-alanine | IAA-Ala | 57105-39-2 | Dr. Ehrenstorfer |
| Indole-3-acetyl-aspartate | IAA-Asp | 2456-73-7 | Dr. Ehrenstorfer |

Table 3 Laboratory equipment.

| Equipment | Model | Brand |
| --- | --- | --- |
| UHPLC | EXIONLC System | SCIEX |
| MS | QP2020 NX | SCIEX |
| Centrifuge | Heraeus Fresco17 | Thermo Fisher Scientific |

1. **Method**
   1. **Metabolites Extraction：**

100 μL aliquot of each individual sample was precisely transferred to an Eppendorf tube. After the addition of 400 μL of extract solution (methanol: acetonitrile = 1: 1, precooled at -40 ℃, containing 0.1% formic acid and isotopically-labelled internal standard mixture), the samples were vortexed for 30 s and sonicated for 10 min in the ice-water bath, followed by subsiding at -40 ℃ for 1h. After centrifugation (15 min, 12000 rpm, and 4 ℃), a 400 *μ*L aliquot of the supernatant was transferred to an Eppendorf tube. Then the supernatant was evaporated to dryness under a gentle stream of nitrogen and was reconstituted in 100 *μ*L water containing 0.1% formic acid. After centrifugation (15 min, 12000 rpm, and 4 ℃), the clear supernatant was subjected to UHPLC-MS/MS analysis.

**2.2 Standard Solution Preparation**

Stock solutions were individually prepared by dissolving or diluting each standard substance to give a final concentration of 1 mmol/L. An aliquot of each of the stock solutions was transferred to an Eppendorf tube form a mixed working standard solution. A series of calibration standard solutions were then prepared by stepwise dilution of this mixed standard solution (containing isotopically-labelled internal standard mixture in identical concentrations with the samples).

**2.3 UHPLC-MRM -MS Analysis：**

The UHPLC separation was carried out using an EXIONLC System (Sciex), equipped with a Waters ACQUITY UPLC HSS T3 column (100 × 2.1 mm, 1.8 *μ*m, Waters). The mobile phase A was 0.1% formic acid in water, and the mobile phase B was 0.1% formic acid in acetonitrile. The column temperature was set at 40 ℃. The auto-sampler temperature was set at 4 ℃ and the injection volume was 5 *μ*L.

A SCIEX 6500 QTRAP+ triple quadrupole mass spectrometer (Sciex), equipped with an IonDrive Turbo V electrospray ionization (ESI) interface, was applied for assay development. Typical ion source parameters were: Curtain Gas = 40 psi, IonSpray Voltage = ±4500 V, temperature = 500 ℃, Ion Source Gas 1 = 30 psi, Ion Source Gas 2 = 30 psi.

The MRM parameters for each of the targeted analytes were optimized using flow injection analysis, by injecting the standard solutions of the individual analytes, into the API source of the mass spectrometer. Several most sensitive transitions were used in the MRM scan mode to optimize the collision energy for each Q1/Q3 pair **(Table 4)**. Among the optimized MRM transitions per analyte, the Q1/Q3 pairs that showed the highest sensitivity and selectivity were selected as ‘quantifier’ for quantitative monitoring. The additional transitions acted as ‘qualifier’ for the purpose of verifying the identity of the target analytes.

SCIEX Analyst Work Station Software (Version 1.6.3) and Sciex MultiQuant software (Version 3.0.3) were employed for MRM data acquisition and processing.

**Table 4 MRM Parameters.**

| **Abbr.** | **Prec Ion** | **Prod Ion** | **Polarity** | **Quantifier/Qualifier** |
| --- | --- | --- | --- | --- |
| 3-HAA | 154 | 136 | Positive | Quantifier |
| 3-HK | 225 | 208 | Positive | Quantifier |
| 5-HIAA | 192 | 146 | Positive | Quantifier |
| 5-HT | 177 | 160 | Positive | Quantifier |
| 5-HTOL | 178 | 160 | Positive | Quantifier |
| 5-HTP | 221 | 204 | Positive | Quantifier |
| 5-Me-IAA | 206 | 160 | Positive | Quantifier |
| AA | 138 | 120 | Positive | Quantifier |
| IA | 188 | 170 | Positive | Quantifier |
| IAA | 176 | 130 | Positive | Quantifier |
| IAM | 175 | 130 | Positive | Quantifier |
| ICA | 146 | 118 | Positive | Quantifier |
| IE | 162 | 144 | Positive | Quantifier |
| IGA | 190 | 144 | Positive | Quantifier |
| ILA | 206 | 118 | Positive | Quantifier |
| Indican | 294 | 294 | Negative | Quantifier |
| Indole | 118 | 118 | Positive | Quantifier |
| IPA | 190 | 130 | Positive | Quantifier |
| IS | 212 | 132 | Negative | Quantifier |
| KYN | 209 | 192 | Positive | Quantifier |
| KYNA | 188 | 144 | Negative | Quantifier |
| Melatonin | 233 | 174 | Positive | Quantifier |
| NA | 124 | 80 | Positive | Quantifier |
| NAS | 219 | 160 | Positive | Quantifier |
| Skatole | 132 | 117 | Positive | Quantifier |
| Trp | 205 | 188 | Positive | Quantifier |
| Tryptamine | 161 | 144 | Positive | Quantifier |
| Xa | 206 | 160 | Positive | Quantifier |
| IAN | 157 | 130 | Positive | Quantifier |
| IAA-Ala | 247 | 130 | Positive | Quantifier |
| IAA-Asp | 291 | 130 | Positive | Quantifier |

**2.4 Calculation formula：**

$$c_{M}\left[ nmol\cdot L^{-1} \right]=\frac{c_{F}\left[ nmol\cdot L^{-1} \right]\cdot V_{F}[\mu L]\cdot CF}{VS[uL]}$$

The final concentration (cF, nmol/L) equals the calculated concentration (cC, nmol/L) multiplied by the dilution factor (Dil). The metabolite concentration (cM, nmol/L) equals the final concentration (cF, nmol/L) multiplied by the final volume (VF, μL) and the sample experiment concentration factor (CF), divided by the sample volume (VS, μL) of the sample. N/A means that the targeted metabolites were not detectable in the corresponding samples.

**Part 4 UHPLC-MS-MS for fecal tryptophan metabolites**

1. **Reagents and equipment**

The reagents and equipment utilized for detecting fecal tryptophan metabolites are identical to those used for detecting serum tryptophan metabolites (**Part 3**).

1. **Method**
   1. **Metabolites Extraction：**

For solid samples, a 50 mg aliquot of each individual sample was precisely weighed and transferred to an Eppendorf tube. After the addition of 500 μL of extract solution (methanol: acetonitrile: H2O = 2: 2: 1, precooled at -40 °C, containing 0.1% formic acid and isotopically-labelled internal standard mixture); the samples were vortexed for 30 s, and homogenized at 35 Hz for 4 min and sonicated for 5 min in ice-water bath. The homogenate and sonicate circle was repeated twice, followed by subsiding at -40 ℃ for 1h. After centrifugation (15 min, 12000 rpm, and 4 ℃), a 320 *μ*L aliquot of the supernatant was transferred to an Eppendorf tube. Then the supernatant was evaporated to dryness under a gentle stream of nitrogen and was reconstituted in 80 *μ*L water containing 0.1% formic acid. After centrifugation (15 min, 12000 rpm, and 4 ℃), the clear supernatant was subjected to UHPLC-MS/MS analysis.

- 1. **Standard Solution Preparation**

The steps for preparing the standard solution are the same as those for detecting serum tryptophan metabolites.

**2.3 UHPLC-MRM -MS Analysis：**

The steps for UHPLC-MRM -MS Analysis are the same as those for detecting serum tryptophan metabolites.

- 1. **Calculation formula：**

$$c_{M}\left[ nmol\cdot{kg}^{-1} \right]=\frac{c_{F}\left[ nmol\cdot L^{-1} \right]\cdot V_{F}\left[ \mu L \right]\cdot CF}{m[mg]}$$

The final concentration (cF, nmol/L) equals the calculated concentration (cC, nmol/L) multiplied by the dilution factor (Dil). The metabolite concentration (cM, nmol/kg) equals the final concentration (cF, nmol/L) multiplied by the final volume (VF, μL) and the sample experiment concentration factor (CF), divided by the mass (m, mg) of the sample.
